# Supplementary material for: A practical concept for catalytic carbonylations using carbon dioxide
Source: Nat Commun. 2022 Jul 30;13:4432. doi: 10.1038/s41467-022-32030-8 (PMC9338997; doi:10.1038/s41467-022-32030-8)
Supplement: Supplementary file 1 — Supplementary Information [file 41467_2022_32030_MOESM1_ESM.pdf]

Supplementary Information

A Practical Concept for Catalytic Carbonylations using Carbon Dioxide

Rui Sang†[1], Yuya Hu†[1], Rauf Razzaq†[1], Guillaume Mollaert[2], Hanan Atia[1], Ursula Bentrup[1], Muhammad Sharif[1], Helfried Neumann[1], Henrik Junge[1], Ralf Jackstell[1]*, Bert Maes[2]*, and Matthias Beller[1]**

[1] *Leibniz-Institut für Katalyse e.V., Albert-Einstein-Str. 29a, 18059 Rostock, Germany*

[2] *Organic Synthesis, Department of Chemistry, University of Antwerp, Groenenborgerlaan 171, 2020 Antwerp, Belgium*

[†] *R. S., Y. H. and R. R. contributed equally to this work.*

*Email: Henrik.junge@catalysis.de; ralf.jackstell@catalysis.de; bert.maes@uantwerpen.be; matthias.beller@catalysis.de

Contents

General information 3
Catalyst preparation for selective generation of CO from CO_2/H_2 in Step I. 5
The Step I catalyst characterization: BET 5
The Step I catalyst characterization: XRD 5
The Step I catalyst characterization: H_2-TPR 6
General procedure for reducing CO_2 with H_2 into CO catalyzed by 10Cu@SiO_2-PHM: Investigation of temperature and pressure. 8
Various carbonylation reactions using CO_2/H_2 as CO source 9
General procedure for hydroformylation of 1a using CO_2/H_2 as CO source: Investigation of ligands. 9
General procedure for hydroformylation of various alkenes using CO_2/H_2 as CO source. 11
General procedure for methoxycarbonylation of 3a using CO_2/H_2 as CO source: Investigation of ligands. 11
General procedure for Pd-catalyzed methoxycarbonylation of various alkynes with CO_2/H_2 as CO source. 12
General procedure for aminocarbonylation of aryl iodide using CO_2/H_2 as CO source 12
Characterization of the products 13
NMR spectra of products 15
Supplementary References 51

General information

Air- and moisture-sensitive synthesis were performed under argon atmosphere in heating gun vacuum dried glassware. Chemicals were purchased from Aldrich, TCI, Alfa, Fluka, Acros or Strem. Unless the purity was less than 97%, all commercial reagents were used without further purification. $Cu(NO_3)_2{\cdot}3H_2O$ (≥99%) salt was obtained from Sigma-Aldrich. Aqueous NH_3 solution (28-30%) was purchased from Roth Chemicals. Silica 60 M was obtained from Macherey-Nagel Germany. CO_2 (99.99%), H_2 (99.99%), Ar (99.99%) and N_2 (99.95%) were provided by Linde Europe. All solvents were degassed prior to use.

The products were characterized by ^{1}H NMR and ^{13}C NMR spectroscopy. ^{1}H and ^{13}C NMR spectra were recorded on Bruker Avance 300 (300 MHz) or 400 (400M) NMR spectrometers. Chemical shifts δ (ppm) are given relative to solvent: references for $CDCl_3$ were 7.26 ppm (^{1}H-NMR) and 77.16 ppm (^{13}C-NMR). ^{13}C-NMR spectra were acquired on a broad band decoupled mode. Multiplets were assigned as s (singlet), d (doublet), t (triplet), dd (doublet of doublet), and m (multiplet).

GC analysis for the gas phase was performed using Agilent HP-PLOT/Q fitted with TCD (thermal conductivity detector) and FID (flame ionization detector) detectors. Carbonylation related GC analysis was performed on a Trace 1310 chromatograph with a 29 m HP5 column. The products were measured by MS and GC analysis or by isolation from the reaction mixture by solvent evaporation and further purified by column chromatography on silica gel.

BET surface area and pore volume of the prepared catalysts were measured from nitrogen adsorption isotherms measured at -196 °C (Micromeritics ASAP 2010). Before the measurement, each sample was degassed at 200 °C for 4 h. The average pore diameters were calculated from the desorption branch of the isotherm using the BJH method. Inductively coupled plasma optical emission spectrometry (ICP-OES) analysis was performed using Varian/Agilent 715-ES analyzer.

XRD powder patterns were recorded on a Stoe STADI P diffractometer, equipped with a linear Position Sensitive Detector (PSD) using Cu K radiation (λ = 1.5406 Å). Processing and assignment of the powder patterns was done using the software WinXpow (Stoe) and the Powder Diffraction File (PDF) database of the International Centre of Diffraction Data (ICDD).

For the TPR experiments, the measurement was done using a Micromeritics Autochem II 2920 instrument. A 100 mg sample was loaded in U shaped quartz reactor and heated from RT to 400°C with 20K/min in 5% O_2/He (50 ml/min) for 30 min at 400 °C, then flushing and cooling down to RT under the flow of Ar. The TPR measurement was carried out from RT to 700 °C (holding time 30 min) in a 5% H_2/Ar flow (50 ml/min) with a heating rate of 10 K/min. Another TPR measurement was done as described previously only in the pretreatment step Ar was used rather than O_2. The hydrogen consumption peaks were recorded with temperature using thermal conductivity detector. Quantitative analysis of the TPR data was calculated based on the peak areas.

The TEM measurements were performed at 200kV with an aberration-corrected JEMARM200F (JEOL, Corrector: CEOS). The microscope is equipped with a JED-2300 (JEOL) energy-dispersive x-ray-spectrometer (EDXS) and an Enfinum ER (GATAN) with Dual EELS for chemical analysis. The solid samples were deposed without any pretreatment on a holey carbon supported Ni-grid (mesh 300) and transferred to the microscope.

XPS data was obtained with a VG ESCALAB220iXL (ThermoScientific) with monochromatic Al Kα (1486.6 eV) radiation. Binding energies were corrected to C-C contribution at 284.8 eV in C1s region. For quantitative analysis, the peaks were deconvoluted with Gaussian-Lorentzian curves, the peak area was divided by a sensitivity factor obtained from the element specific Scofield factor and the transmission function of the spectrometer.

For the characterization of the Cu species CO was used as probe molecule. In situ FTIR spectroscopic measurements in transmission mode were carried out on a Bruker Tensor 27 FTIR spectrometer equipped with a heatable and evacuable homemade reaction cell with CaF_2 windows connected to a gas-dosing and evacuation system. The sample powders were pressed into self-supporting wafers with a diameter of 20 mm and a weight of 50 mg. The samples were pretreated by heating in vacuum up to 400°C and keeping at this temperature for 1h. After dosing CO_2/H_2=1:3 for 5 min the reaction cell was closed, and the reaction was monitored for 60 min. After cooling to room temperature and evacuation the sample was exposed to 5% CO/He. The CO adsorbate spectrum was recorded after removing the gas phase by evacuation the cell.

The reaction set-up was shown in Fig. S1.

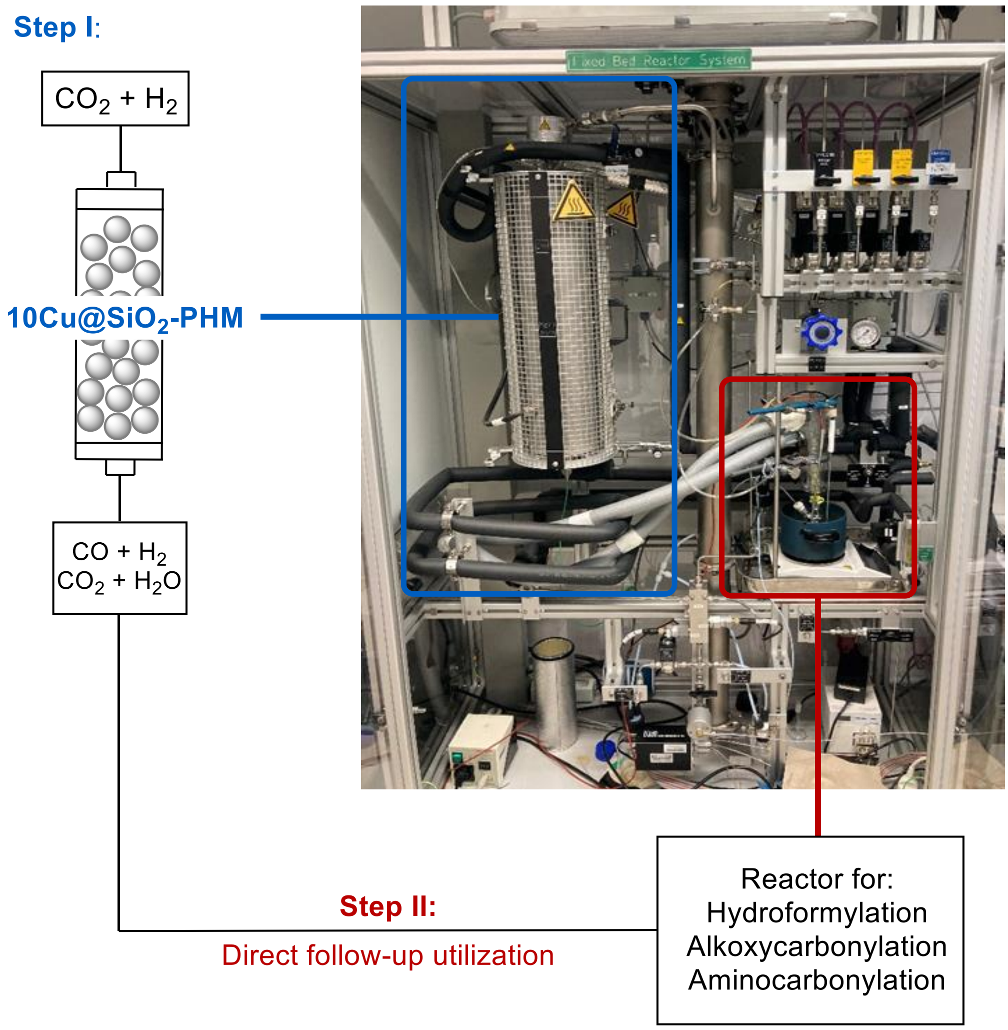

Fig. S1 The mini plant for catalytic carbonylations using carbon dioxide. The flow reactor for the selective CO generation from CO_2/H_2 catalyzed by 10Cu@SiO_2-PHM, and the cascade direct utilization of generated CO in various carbonylation reactions.

Catalyst preparation for selective generation of CO from CO_2/H_2 in Step I.

The catalysts Cu@supports-PHM were prepared using precipitation-hydrothermal method (PHM). In a typical synthesis process, desired amount of metal precursor was dissolved in 20 mL of DI H_2O to obtain different metal loadings and stirred for 15 min. 2.5 ml of 28-30% Aq. NH_3 solution was added dropwise under continuous stirring for 30 min. Then the support (SiO_2, Al_2O_3, or C) was added to the precipitate and the mixture was stirred vigorously for 4 h at room temperature. The contents were then transferred to a 100 mL autoclave and heated at 120 °C for 8 h without stirring. The autoclave was then allowed to cool naturally to room temperature and the slurry was centrifuged and washed several times with ethanol and dried at 80 °C for 10 h. The dried material was then pyrolysed at 600 °C for 2 h under Ar atmosphere. For comparison, the catalyst 10Cu@SiO_2-CIM was also prepared using conventional impregnation method. Here the silica support was directly added to metal nitrate solution and stirred for 4 h followed by drying at 80 °C for 10 h and pyrolysis in Ar at 600 °C for 2 h.

The Step I catalyst characterization: BET

The physiochemical properties of all the prepared and tested catalysts were studied using ICP and BET analysis as listed in Table S1. The ICP analysis showed that the actual metal loading was close to the initial designed value. The surface area of all the samples decreased upon metal loading as compared to blank silica support which was understandable due to the blockage of pores by metal particles which was also evident from the decrease in the total pore volume.

Table S1. **Physiochemical Properties of the prepared catalysts.**

Entry	Catalysts	(Cu)[a] (wt%)	S_{BET} (m^2/g)	TPV (cm^3/g)	DP (nm)
1	SiO_2	-	546	0.86	6.3
2	5Cu@SiO_2-PHM	4.6	415	0.75	8.2
3	10Cu@SiO_2-PHM	9.1	370	0.67	7.5
4	10Cu@SiO_2-CIM	8.3	433	0.71	6.5

[a] Determined by ICP-OES

The Step I catalyst characterization: XRD

The XRD analysis was used to study the characteristics of different crystalline and amorphous phases of the samples (Fig. S2). The blank silica support had a highly amorphous phase with a broad peak at $2\theta = 22.5°$ (JCPDS database PDF: 00-039-1425, SiO_2-cristobalite). The XRD pattern of the 10CIM sample showed diffraction peak of highly crystalline CuO (tenorite JCPDS: 00-041-0254) indicating the formation of large metal particles.[1-2] On the contrary, the sample prepared using PHM had no CuO peak which indicates that it is either present in the reduced state (Cu_2O/Cu^0) or is highly dispersed over the support with small nanoparticles which could not be detected using XRD analysis. Moreover, two other distinct metallic phases of Cu_2O (cubic JCPDS: 01-071-3645) and Cu^0 (cubic JCPDS: 01-071-4610) were observed.[3-4]

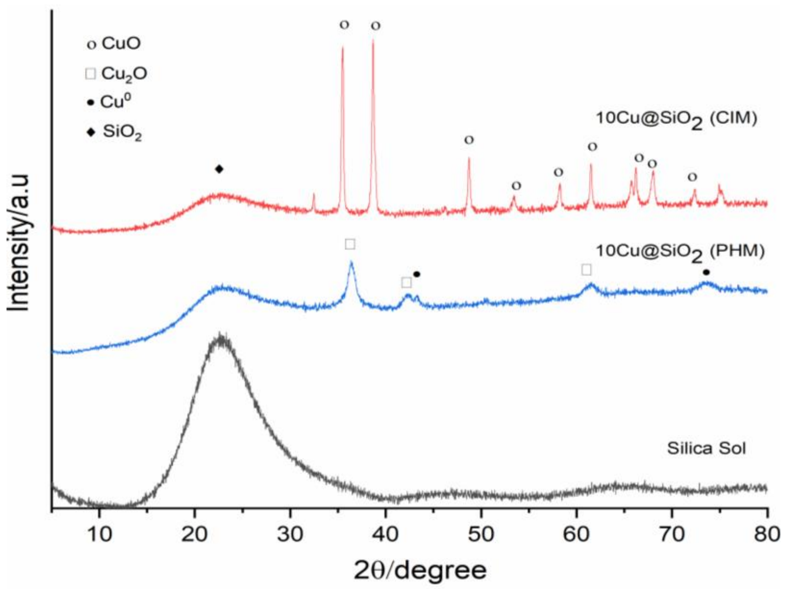

Fig. S2 Catalysts XRD characterization. XRD patterns of SiO_2, 10Cu@SiO_2-PHM, and 10Cu@SiO_2-CIM samples.

The Step I catalyst characterization: H_2-TPR

The H_2-TPR (Fig. S3a) was performed to study the metal oxide reducibility of the different prepared samples after pretreatment under 5% O_2/He. The 10Cu@SiO_2-PHM sample showed a single low temperature reduction peak indicating the presence of only one type of finely dispersed CuO species with no large CuO clusters. On the contrary, the 10Cu@SiO_2-CIM sample has two distinct peaks which can be assigned to two different types of Cu species. The low temperature reduction peak at 250 °C can be attributed to smaller and well dispersed CuO, while a broad high temperature reduction peak at 410 °C can be ascribed to larger and poorly dispersed CuO crystallites. It has been previously established that Cu^{2+} species strongly interacting with the support are more difficult to be reduced as compared to CuO in bulk.[5-8] The results are in well agreement to the XRD analysis of CIM catalyst which shows the formation of CuO with large crystalline size. To further identify the nature of Cu species in our samples, TPR experiments (Fig. S3b-c) were performed using Argon pre-treatment (30 min at 200 °C) instead of O2. Then the sample was cooled down to room temperature followed by TPR analysis by heating the sample under the flow of 5%H_2/Ar, 10K/min up to 700 °C and kept for 30 min at 700 °C. From these measurements, the experimental amount of H2 consumption of an inactive 10Cu@SiO2-CIM sample was 1290.8 µmol/g (ICP = 8.3 is 1307 µmol/g), indicating that it contains only Cu^{2+} which was almost completely reduced to Cu0 at higher temperature (390 °C). However, our active 10Cu@SiO_2-PHM showed 627.97 µmol/g (ICP = 9.1 is equal to 1433.1 µmol/g) H_2 consumption which indicates the presence of a mixture of Cu^{1+}/Cu^{2+}. Additionally, the active sample reduction profile starts at lower temperature (104°C, Fig. S3b) in comparison with 10Cu@SiO_2-CIM which only shows a minor hump at the lower reduction temperature.

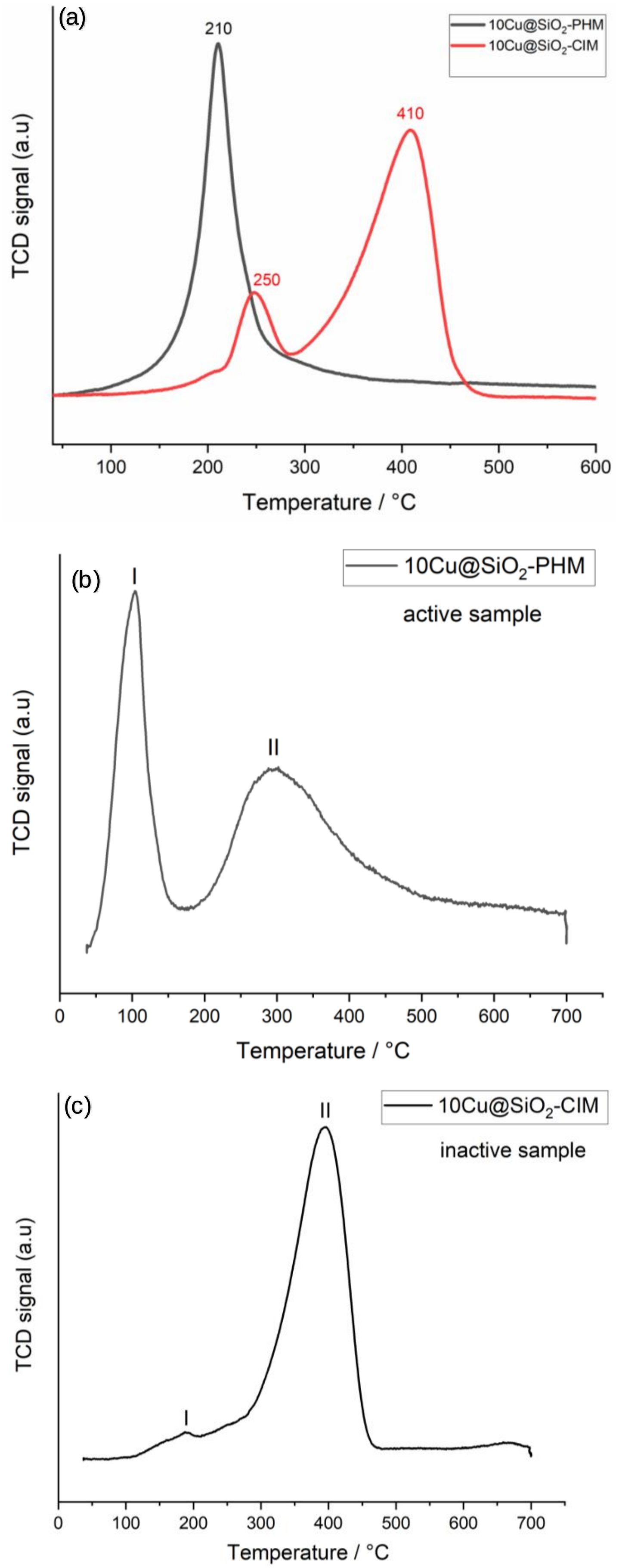

Fig. S3 H_2-TPR profile of 10Cu@SiO_2-PHM and 10Cu@SiO_2-CIM samples. a) O_2 pre-treatment **b**) Ar pre-treatment (active sample) **c**) Ar pre-treatment (inactive sample)

General procedure for reducing CO_2 with H_2 into CO catalyzed by 10Cu@SiO_2-PHM: Investigation of temperature and pressure.

The reaction for reducing CO_2 with H_2 into CO was carried out in a fixed bed flow-reactor (8.9 mm ID). The catalyst bed at the center of the reactor was supported by glass wool and quartz sand (dilution 1:5). Temperature was measured by a K-type thermocouple inserted into the catalyst bed.

A gas mixture of H_2 and CO_2 (H_2:CO_2= 3:1) at a total flow rate of 100 NmL/min was fed into the reactor (0.3 g catalyst, 0.25–0.42 mm, GHSV = 15,000 h^{-1}). The volumetric flow rate of the feed gases was controlled by pre-calibrated mass flow controllers (Brooks Instrument). The reaction temperature was increased from 200 to 400°C. The CO_2 conversion and product selectivity are defined as follows:

$$Conversion = 100 \times mCO_{2(in)} - mCO_{2(out)} \Big/ mCO_{2(in)} \qquad (1)$$

$$Selectivity = 100 \times m_{product_{(out)}\times carbon\,number} \Big/ mCO_{2(in)} - mCO_{2(out)} \qquad (2)$$

where $mCO_{2(in)}$ and $mCO_{2(out)}$ are the moles of CO_2 in and out of the reactor. The selectivity is defined as the percentage of moles of CO_2 consumed to form desired product (CO, CH_4 or MeOH), in respect to the amount of CO_2 consumed during the reaction.

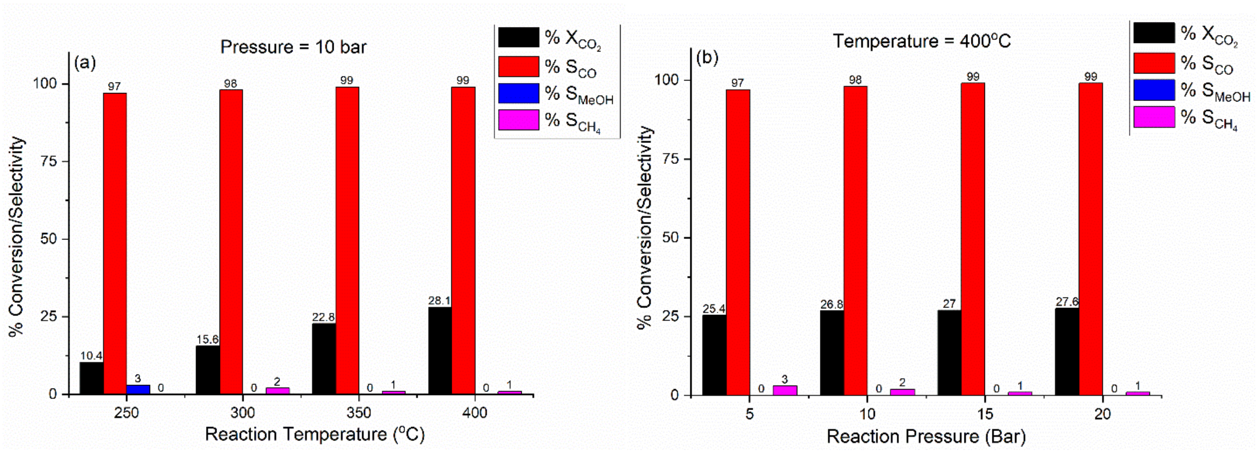

Fig. S4 Effect of different reaction conditions using 10Cu@SiO_2-PHM catalyst in flow-reactor for CO_2 hydrogenation experiment. a) The effect of temperature. **b**) The effect of pressure.

Various carbonylation reactions using CO_2/H_2 as CO source.

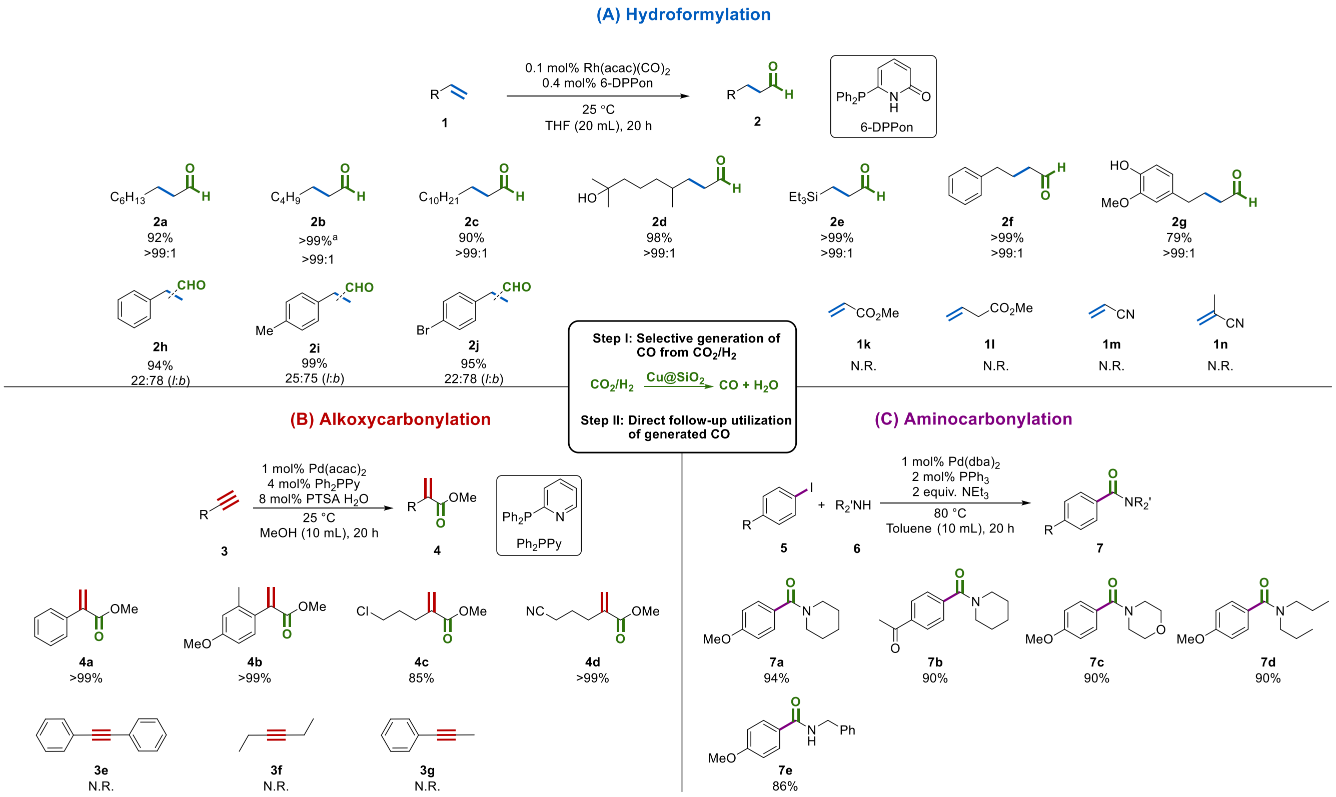

Fig. 5 Various carbonylation reactions using CO_2/H_2 as CO source. Cu-catalyzed selective CO_2 to CO conversion and the follow-up utilization in carbonylation reactions. (N.R.: no reaction)

General procedure for hydroformylation of 1a using CO_2/H_2 as CO source: Investigation of ligands.

Under argon atmosphere, a 25 mL three-necked round bottom flask was charged with $Rh(acac)(CO)_2$ (0.1 mol%), monodentate ligand (0.4 mol%) or bidentate ligand (0.2 mol%), and oven dried stirring bar. The flask was assembled with a -20°C condenser and a pipeline which connect to the fixed bed flow-reactor. The water generated during the CO_2 reduction (step I) was not removed and the resulting gas mixture (CO_2, H_2, CO) was constantly bubbled through the reaction solution at flow-rate of 100 ml/min. **1a** (10 mmol, 1.6 mL) and THF (20.0 mL) were injected into the flask by syringe. The flask was then sealed with cap, the condenser was connected to the ventilation system and the flow-reactor was opened for bubbling in the solution. The GC analysis was performed while taking a gas sample directly from the reaction flask. Gas composition (%) analysis by GC of the gas mixture (H_2 : CO : CO_2 = 70 : 9.0 : 21). The reaction was performed for 20 h at 25 °C. After the reaction finished, the yield and *l*/*b* selectivity were determined by GC analysis using isooctane as the internal standard.

Table S2: Hydroformylation of 1a using CO_2/H_2 as CO source: ligand investigation.

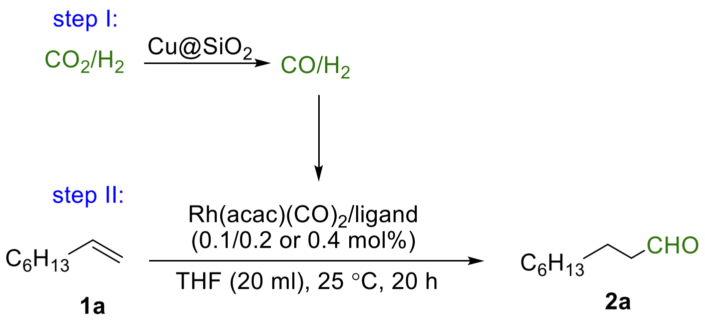

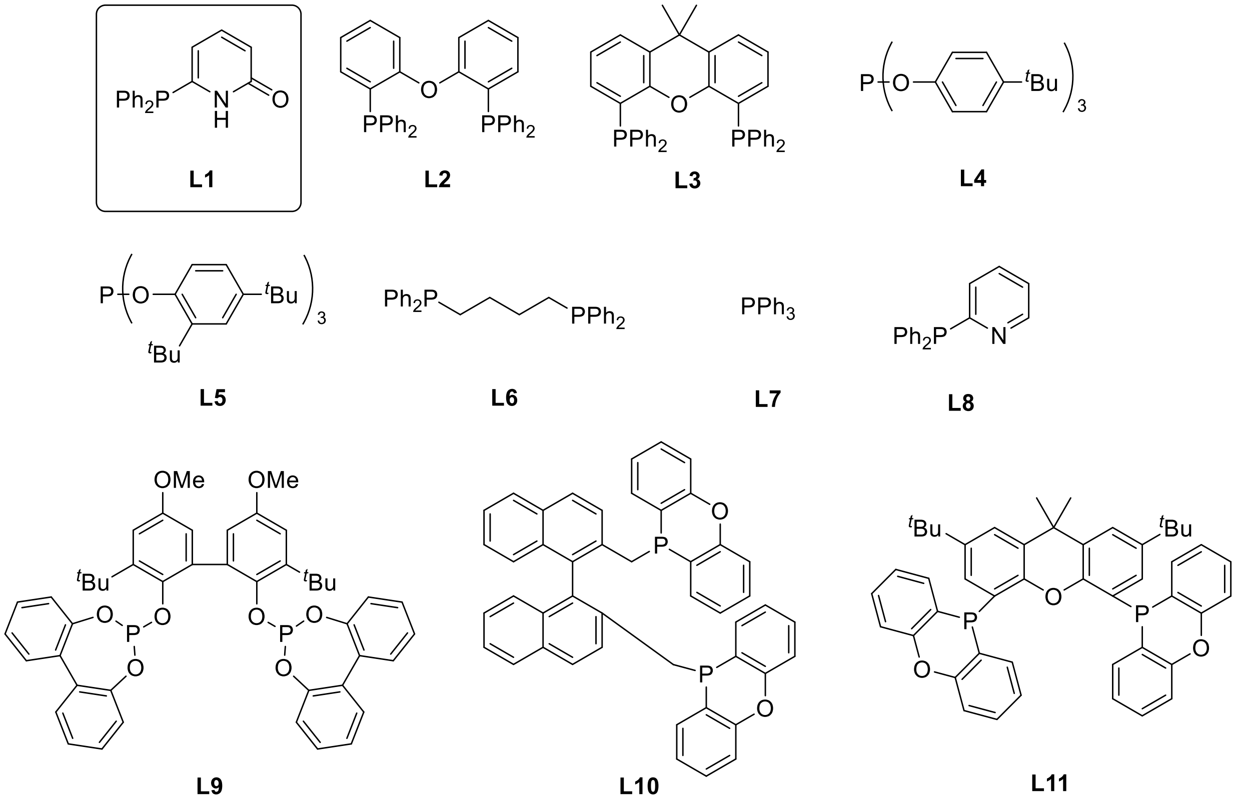

Entry	L	**1a** conversion/%	**2a** yield/%(*l*/*b*)	Isomers of **1a**
1	**L1**	91	85 (98/2)	6
2	**L2**	8	6 (83/17)	2
3	**L3**	0	-	-
4	**L4**	11	8 (96/4)	3
5	**L5**	0	-	-
6	**L6**	0	-	-
7	**L7**	0	-	-
8	**L8**	55	54 (81/19)	1
9	**L9**	99	54 (85/15)	45
10	**L10**	4	2 (>99/1)	2
11	**L11**	35	27 (>99/1)	8
12[a]	**L1**	>99	92 (>99/1)	6

Reaction conditions: **1a** (10 mmol), $Rh(acac)(CO)_2$ (0.1 mol%), bidentate (0.2 mol%) or monodentate (0.4 mol%), THF (20 mL), 25 °C, gas outlet from the fixed bed flow-reactor, 20 h. The yield and selectivity were determined by GC analysis. [a]Using 5 mmol of **1a**.

General procedure for hydroformylation of various alkenes using CO_2/H_2 as CO source.

Under argon atmosphere, a 25 mL three-necked round bottom flask was charged with Rh(acac)$(CO)_2$ (1.3 mg, 0.1 mol%), 6-diphenylphosphino-2-pyridone (**L1**, 5.6 mg, 0.4 mol%), and oven dried stirring bar. The flask was assembled with a -20°C condenser and a pipeline which connect to the fixed bed flow-reactor. Alkene (5.0 mmol) and THF (20.0 mL) were injected into the flask by syringe. The flask was then sealed with cap, the condenser was connected to the ventilation system and the flow-reactor was opened for bubbling in the solution. The reaction was performed for 20 h at 25 °C. After the reaction finished, the *l*/*b* selectivity was determined by GC analysis, and the product was isolated with column chromatography.

General procedure for methoxycarbonylation of 3a using CO_2/H_2 as CO source: Investigation of ligands.

Under argon atmosphere, a 25 mL three-necked round bottom flask was charged with Pd$(acac)_2$ (15 mg, 1.0 mol%), monodentate ligand (4.0 mol%) or bidentate ligand (2.0 mol%), *para*-toluenesulfonic acid monohydrate (PTSA▪H_2O, 76 mg, 8.0 mol%) and oven dried stirring bar. The flask was assembled with a -20°C condenser and a pipeline which connect to the fixed bed flow-reactor. Phenylacetylene (**3a**, 0.55 mL, 5.0 mmol), and MeOH (10.0 mL) were injected into the flask by syringe. The flask was then sealed with cap, the condenser was connected to the ventilation system and the flow-reactor was opened for bubbling in the solution. The reaction was performed at 25 °C. After 20 h, the reaction was stopped, the yields and selectivity were determined by GC using isooctane as internal standard.

Table S3: Methoxycarbonylation of 3a using CO_2/H_2 as CO source: Investigation of ligands.

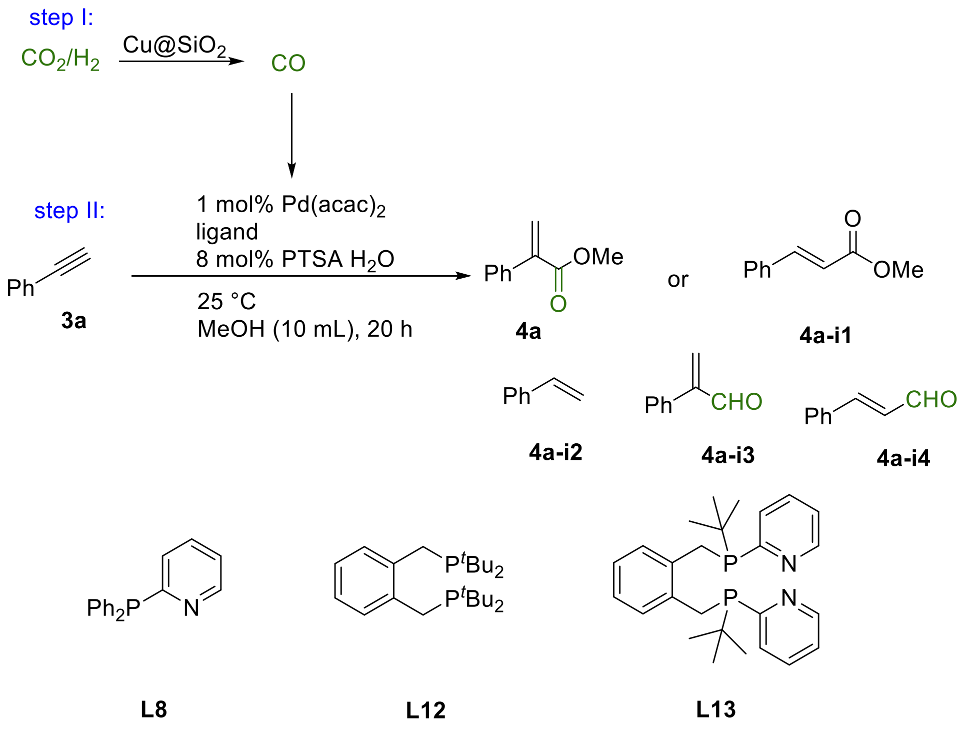

Entry	ligand	Conversion/%	4a/%	4a-i1/%	4a-i2/%	4a-i3/%	4a-i4/%
1	**L8**	>99	99	-	-	-	-
2	**L12**	50	21	3	50	8	18
3	**L13**	>99	-	-	16	53	31
4[a]	**L13**	2	-	-	-	2	-

Reaction condition: $Pd(acac)_2$ (15 mg, 1.0 mol%), **Ligand** (2.0 or 4.0 mol%), PTSA▪H_2O (76 mg, 8.0 mol%), **3a** (0.55 mL, 5 mmol), MeOH (10 mL), gas outlet from the fixed bed flow-reactor, 25 °C. The yield and ratios of products were determined by GC analysis. [a]Toluene (10 mL), MeOH (0.83 mL, 4 equiv., 20 mmol).

General procedure for Pd-catalyzed methoxycarbonylation of various alkynes with CO_2/H_2 as CO source.

Under argon atmosphere, a 25 mL three-necked round bottom flask was charged with $Pd(acac)_2$ (15 mg, 1.0 mol%), 2-(diphenylphosphino)pyridine (**L8**, 53 mg, 4.0 mol%), *para*-toluenesulfonic acid monohydrate (PTSA▪H_2O, 76 mg, 8.0 mol%) and oven dried stirring bar. The flask was assembled with a -20°C condenser and a pipeline which connect to the fixed bed flow-reactor. Alkynes (**3**, 5.0 mmol), and MeOH (10.0 mL) were injected into the flask by syringe. The flask was then sealed with cap, the condenser was connected to the ventilation system and the flow-reactor was opened for bubbling in the solution. The reaction was performed at 25 °C. After 20 h, the reaction was stopped, the selectivity was determined by GC analysis, and the product was isolated with column chromatography.

General procedure for aminocarbonylation of aryl iodide using CO_2/H_2 as CO source.

Under argon atmosphere, a 25 mL two-necked round bottom flask was charged with $Pd(dba)_2$ (17 mg, 0.03 mmol, 1.0 mol%), PPh_3 (16 mg, 0.06 mmol, 2.0 mol%), aryl iodide (3 mmol), and oven dried stirring bar. The flask was assembled with a -20°C condenser and a pipeline connected to the fixed bed flow-reactor. Toluene (10 mL), Et_3N (0.84 mL, 6.0 mmol, 2.0 equiv.) and amine (6.0 mmol, 2.0 equiv.) were injected into the flask by syringe. The flask was then sealed with cap, the condenser was connected to the ventilation system and the flow-reactor was opened for bubbling in the solution. The reaction was stirred for 20 h at 80 °C. After 20 h, the conversion and selectivity were determined by GC analysis, and the product was isolated with column chromatography.

Characterization of the products

1-Nonanal (**2a**): ^{1}H NMR (300 MHz, $CDCl_3$): δ = 9.71 (s, 1H), 2.40–2.35 (m, 2H), 1.61–1.54 (m, 2H), 1.25–1.23 (m, 10H), 0.86–0.82 (t, 3H). ^{13}C NMR (75 MHz, $CDCl_3$): δ = 202.65, 43.78, 31.68, 29.21, 29.06, 28.98, 22.51, 21.97, 13.92.

1-Tridecanal (**2c**): ^{1}H NMR (300 MHz, $CDCl_3$): δ = 9.75 (s, 1H), 2.43–2.38 (td, *J* = 7.4, 1.9 Hz, 2H), 1.66–1.57 (q, *J* = 7.2 Hz, 2H), 1.28–1.25 (m, 19H), 0.89–0.84 (m, 3H). ^{13}C NMR (75 MHz, $CDCl_3$): δ = 202.79, 43.86, 31.87, 29.60, 29.58, 29.54, 29.39, 29.32, 29.30, 29.12, 22.64, 22.04, 14.04.

8-Hydroxy-4,8-dimethylnonanal (**2d**): ^{1}H NMR (300 MHz, $CDCl_3$): δ = 9.77 (t, 1H), 2.46–2.39 (m, 2H), 1.83 (br, 1H), 1.72–1.62 (m, 2H), 1.50–1.26 (m, 8H), 1.20 (s, 6H), 0.90–0.88 (d, *J*= 6.4 Hz, 3H). ^{13}C NMR (75 MHz, $CDCl_3$): δ = 202.92, 71.01, 44.03, 41.66, 37.14, 32.34, 29.26, 29.18, 28.82, 21.62, 19.27.

3-(Triethylsilyl)propanal (**2e**): ^{1}H NMR (400 MHz, $CDCl_3$): δ = 9.71 (s, 1H), 2.36–2.32 (m, 2H), 0.93–0.89 (t, *J* = 8.0 Hz, 9H), 0.77–0.73 (m, 2H), 0.54–0.48 (q, *J* = 8.0 Hz, 6H). ^{13}C NMR (100 MHz, $CDCl_3$): δ = 202.93, 38.22, 7.18, 2.99.

4-Phenylbutanal (**2f**): ^{1}H NMR (300 MHz, $CDCl_3$): δ = 9.78 (s, 1H), 7.36–7.20 (m, 5H), 2.72–2.67 (t, 2H), 2.51–2.46 (m, 2H), 2.05–1.95 (m, 2H). ^{13}C NMR (75 MHz, $CDCl_3$): δ = 202.13, 141.13, 128.36, 126.00, 43.02, 34.90, 23.56.

4-(4-Hydroxy-3-methoxyphenyl)butanal (**2g**): ^{1}H NMR (300 MHz, $CDCl_3$): δ = 9.77 (t, 1H), 6.86–6.83 (d, *J* = 8.5 Hz 1H), 6.68–6.64 (d, *J* = 6.8 Hz, 2H), 5.50 (br, 1H), 3.89 (s, 1H), 2.62–2.57 (m, 2H), 2.49–2.43 (m, 2H), 1.99–1.89 (m, 2H). ^{13}C NMR (75 MHz, $CDCl_3$): δ = 202.43, 143.89, 133.12, 121.02, 114.24, 110.93, 55.86, 43.11, 34.70, 23.91. HRMS (EI) calculated for $C_{11}H_{14}O_3$ [M]: 194.09375; found: 194.09335.

2-Phenylpropanal (**2h**): ^{1}H NMR (400 MHz, $CDCl_3$): δ = 9.71 (s, 1H), 7.42–7.38 (m, 2H), 7.34–7.30 (m, 1H), 7.24–7.7.22 (m, 2H), 3.68–3.62 (q, *J* = 7.3 Hz, 1H), 1.47–1.45 (d, *J* = 7.1 Hz, 3H). ^{13}C NMR (100 MHz, $CDCl_3$): δ = 201.19, 137.69, 129.06, 128.30, 127.50, 52.99, 14.58. HRMS (EI) calculated for $C_9H_{10}O$ [M]: 134.07262; found: 134.07264.

2-(*p*-Tolyl)propanal (**2i**): ^{1}H NMR (400 MHz, $CDCl_3$): δ = 9.68 (s, 1H), 7.22–7.20 (d, *J* = 7.7 Hz, 2H), 7.13–7.11 (d, *J* = 8.1 Hz, 2H), 3.64–3.58 (q, *J* = 7.3 Hz, 1H), 2.36 (s, 3H), 1.45 – 1.43 (d, *J* = 7.0 Hz, 3H). ^{13}C NMR (100 MHz, $CDCl_3$): δ = 201.21, 137.24, 134.61, 129.76, 128.18, 52.60, 21.03, 14.60. HRMS (EI) calculated for $C_{10}H_{12}O$ [M]: 148.08827; found: 148.08789.

2-(4-Bromophenyl)propanal (**2j**): ^{1}H NMR (400 MHz, $CDCl_3$): δ = 9.71 (s, 1H), 7.57–7.54 (d, *J* = 8.5 Hz, 2H), 7.15–7.13 (d, *J* = 8.1 Hz, 2H), 3.69–3.64 (q, *J* = 7.4 Hz, 1H), 1.49–1.48 (d, *J* = 7.1 Hz, 3H). ^{13}C NMR (100 MHz, $CDCl_3$): δ = 200.30, 136.62, 132.10, 129.92, 121.50, 52.29, 14.50. HRMS (EI) calculated for C_9H_9BrO [M]: 211.98313; found: 211.98282.

Methyl 2-phenylacrylate (**4a**): ^{1}H NMR (300 MHz, $CDCl_3$): δ = 7.46–7.36 (m, 5H), 6.39 (d, *J* = 1.2 Hz, 1H), 5.92 (d, *J* = 1.2 Hz, 1H), 3.85 (s, 3H). ^{13}C NMR (75 MHz, $CDCl_3$): δ = 167.14, 141.19, 136.60, 128.18, 128.08, 128.01, 126.79, 52.08. HRMS (EI) calculated for $C_{10}H_{10}O_2$ [M]: 162.06753; found: 162.06765.

Methyl 2-(4-methoxy-2-methylphenyl)acrylate (**4b**): ^{1}H NMR (400 MHz, $CDCl_3$): δ = 7.09–7.07 (d, 1H), 6.76–6.72 (m, 2H), 6.48 (s, 1H), 5.69 (s, 1H), 3.81 (s, 3H), 3.77 (s, 3H), 2.19 (s, 3H). ^{13}C NMR (75 MHz, $CDCl_3$): δ = 167.49, 159.37, 141.23, 137.60, 130.58, 129.70, 128.42, 115.47, 110.82, 55.16, 52.19, 20.09. HRMS (EI) calculated for $C_{12}H_{14}O_3$ [M]: 206.09375; found: 206.09412.

Methyl 5-chloro-2-methylenepentanoate (**4c**): ^{1}H NMR (400 MHz, $CDCl_3$): δ = 6.21 (s, 1H), 5.62 (s, 1H), 3.77 (s, 3H), 3.57–3.54 (t, 2H), 2.50–2.46 (t, *J*= 7.4 Hz, 2H), 2.00–1.93 (m, 2H). ^{13}C NMR (100 MHz, $CDCl_3$): δ = 167.32, 138.91, 125.99, 51.88, 44.17, 31.08, 29.23.

Methyl 5-cyano-2-methylenepentanoate (**4d**): ^{1}H NMR (300 MHz, $CDCl_3$): δ = 6.23 (s, 1H), 5.63 (s, 1H), 3.76 (s, 3H), 2.49–2.44 (m, 2H), 2.38–2.33 (m, 2H), 1.91–1.15 (m, 2H). ^{13}C NMR (75 MHz, $CDCl_3$): δ = 167.32, 138.44, 126.79, 119.43, 52.11, 31.12, 24.30, 16.65. HRMS (ESI) calculated for $C_8H_{11}NO_2$ $[M+Na]^+$: 176.0687; found: 176.0682.

(4-Methoxyphenyl)(piperidin-1-yl)methanone (**7a**): ^{1}H NMR (400 MHz, $CDCl_3$): δ = 7.36–7.33 (m, 2H), 6.89–6.87 (m, 2H), 3.80 (s, 3H), 3.51 (bs, 4H), 1.66–1.56 (m, 6H). ^{13}C NMR (100 MHz, $CDCl_3$): δ = 170.16, 160.38, 128.72, 113.49, 55.19, 48.10, 43.38, 25.95, 24.51.

1-(4-(Piperidine-1-carbonyl)phenyl)ethan-1-one (**7b**): ^{1}H NMR (300 MHz, $CDCl_3$): δ = 8.00–7.96 (d, *J*= 8.6 Hz, 2H), 7.49–7.45 (d, *J*= 8.5 Hz 2H), 3.71 (bs, 2H), 3.29 (bs, 2H), 2.61 (s, 3H), 1.70–1.66 (m, 4H), 1.51 (bs, 2H). ^{13}C NMR (75 MHz, $CDCl_3$): δ = 197.38, 169.06, 140.91, 137.47, 128.43, 126.91, 48.57, 43.03, 26.64, 26.45, 25.51, 24.43. HRMS (ESI) calculated for $C_{14}H_{17}NO_2$ $[M+H]^+$: 232.1337; found: 232.1339.

(4-Methoxyphenyl)(morpholino)methanone (**7c**): ^{1}H NMR (300 MHz, $CDCl_3$): δ = 7.36–7.31 (m, 2H), 6.89–6.84 (m, 2H), 3.77 (s, 3H), 3.64–3.53 (m, 8H). ^{13}C NMR (75 MHz, $CDCl_3$): δ = 170.20, 160.68, 128.98, 127.06, 113.57, 66.66, 55.14, 47.15, 43.26.

4-Methoxy-*N*,*N*-dipropylbenzamide (**7d**): ^{1}H NMR (300 MHz, $CDCl_3$): δ = 7.33–7.31 (m, 1H), 7.30–7.28 (m, 1H), 3.80 (s, 3H), 3.30 (br, 4H), 1.59 (br, 4H), 0.84 (br, 6H). ^{13}C NMR (75 MHz, $CDCl_3$): δ = 171.60, 160.09, 129.53, 128.24, 113.51, 56.18, 50.40, 46.34, 21.22, 11.13. HRMS (ESI) calculated for $C_{14}H_{21}NO_2$ $[M+H]^+$: 236.1650; found: 236.1651.

***N*-Benzyl-4-methoxybenzamide** (**7e**): ^{1}H NMR (300 MHz, $CDCl_3$): δ = 7.79–7.78 (m, 1H), 7.76–7.75 (m, 1H), 7.37–7.30 (m, 5H), 6.95–6.93 (m, 1H), 6.91–6.90 (m, 1H), 6.38 (br, 1H), 4.65–4.63 (d, 2H), 3.85 (s, 3H). ^{13}C NMR (75 MHz, $CDCl_3$): δ = 166.82, 162.20, 138.37, 128.74, 127.89, 127.53, 126.61, 113.74, 55.38, 44.05. HRMS (ESI) calculated for $C_{15}H_{15}NO_2$ $[M+H]^+$: 242.1181; found: 242.1183.

NMR spectra of products

^{1}H NMR of 1-Nonanal (2a)

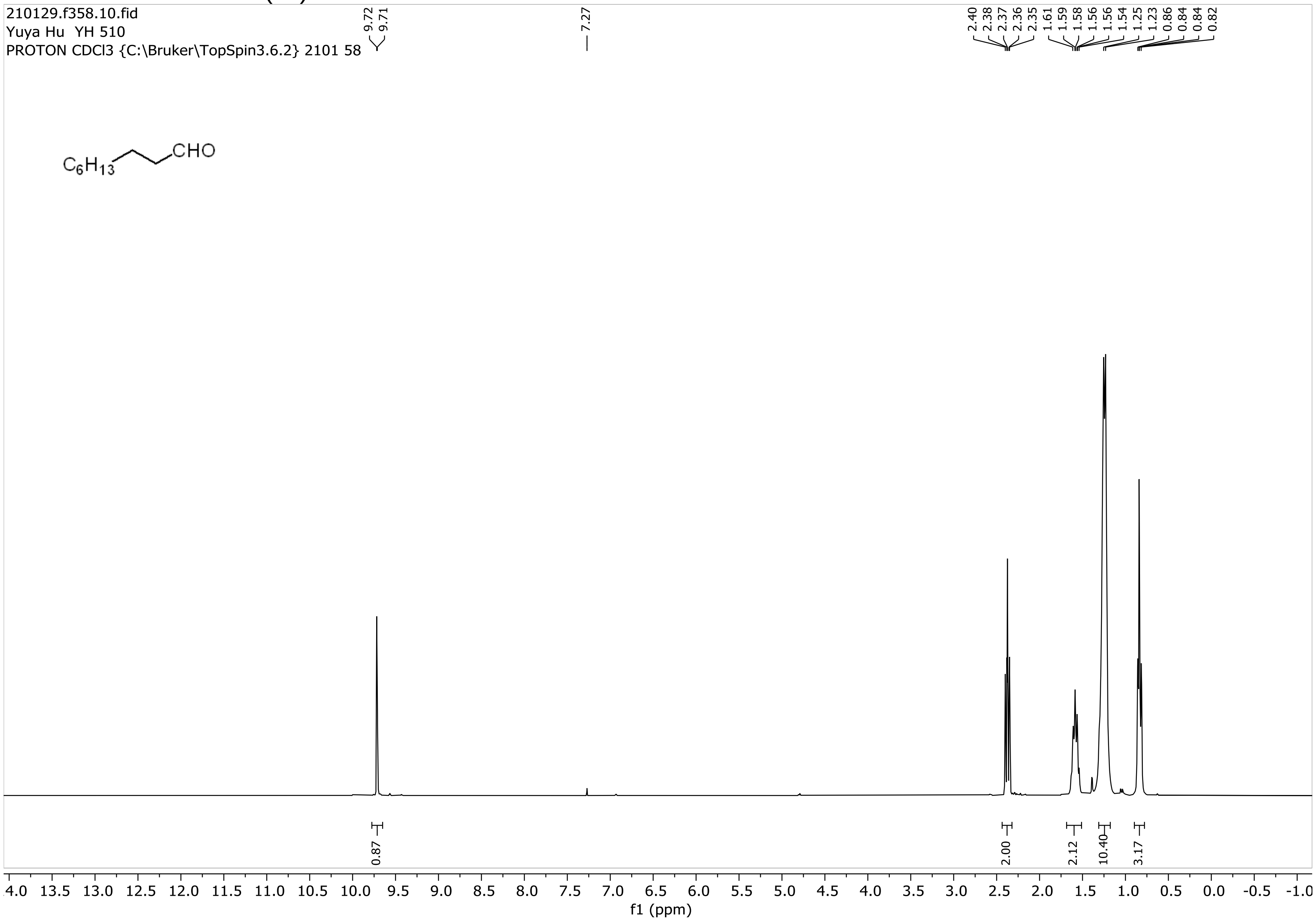

^{13}C NMR of 1-Nonanal (2a)

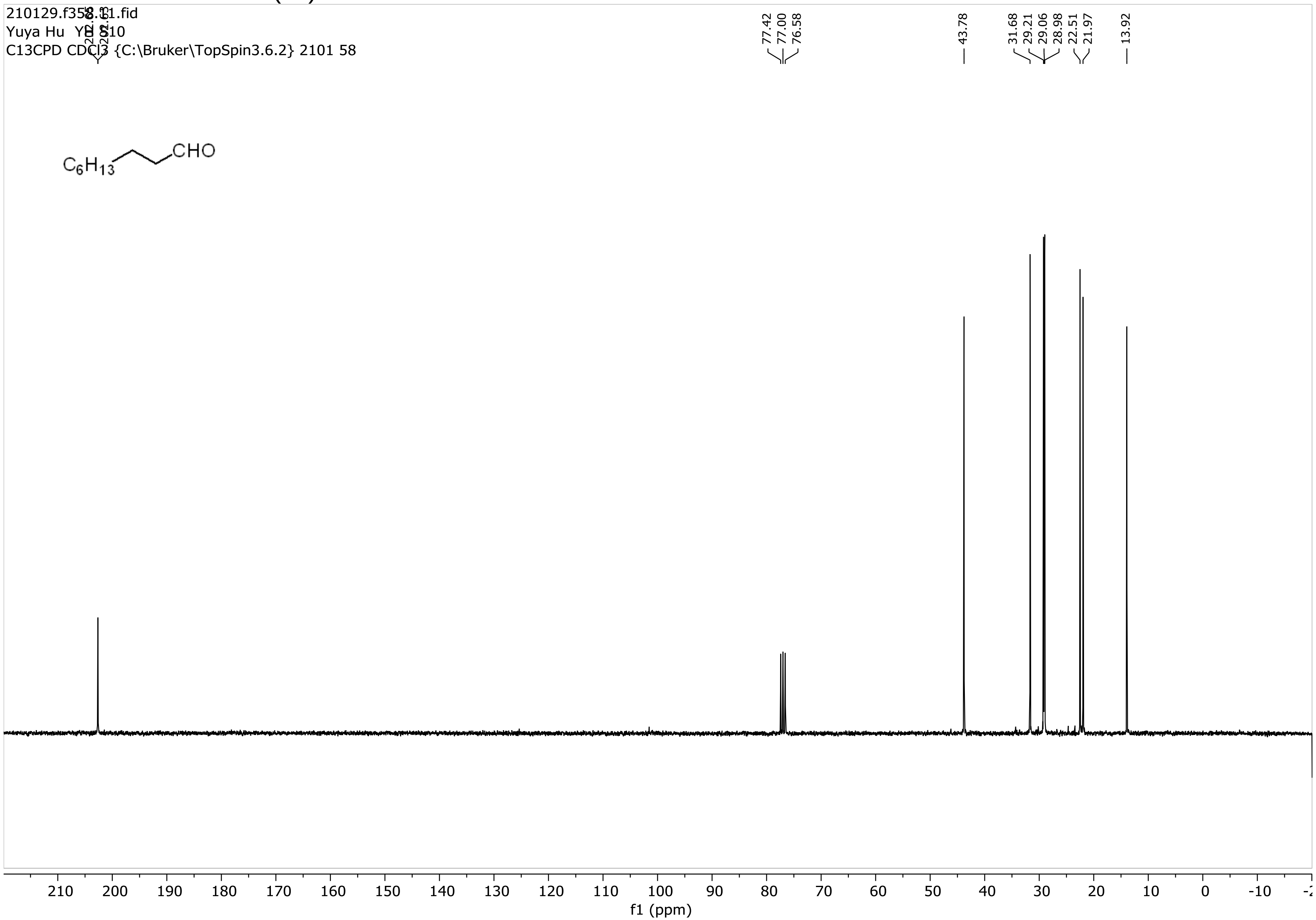

^{1}H NMR of 1-Tridecanal (2c)

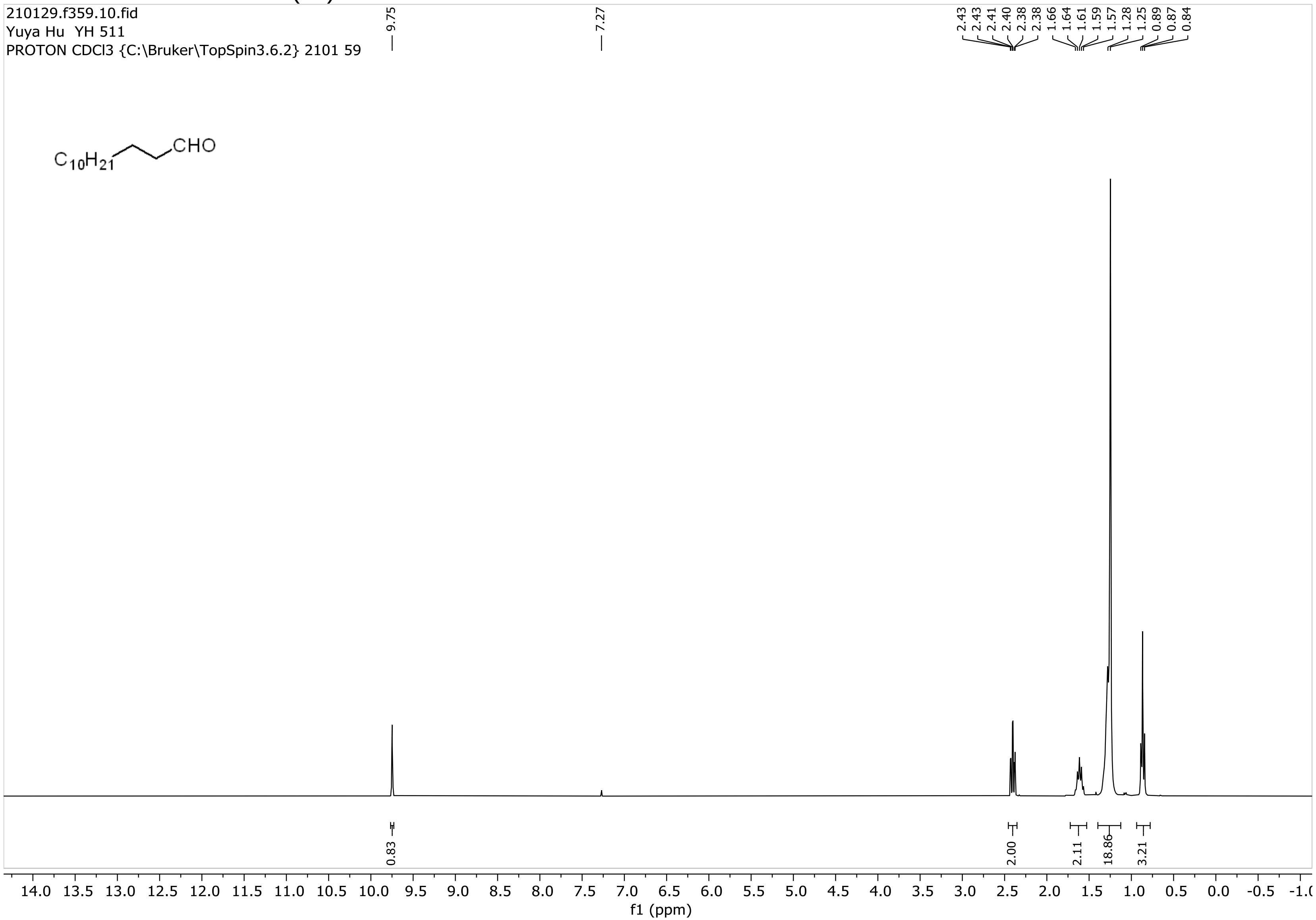

^{13}C NMR of 1-Tridecanal (2c)

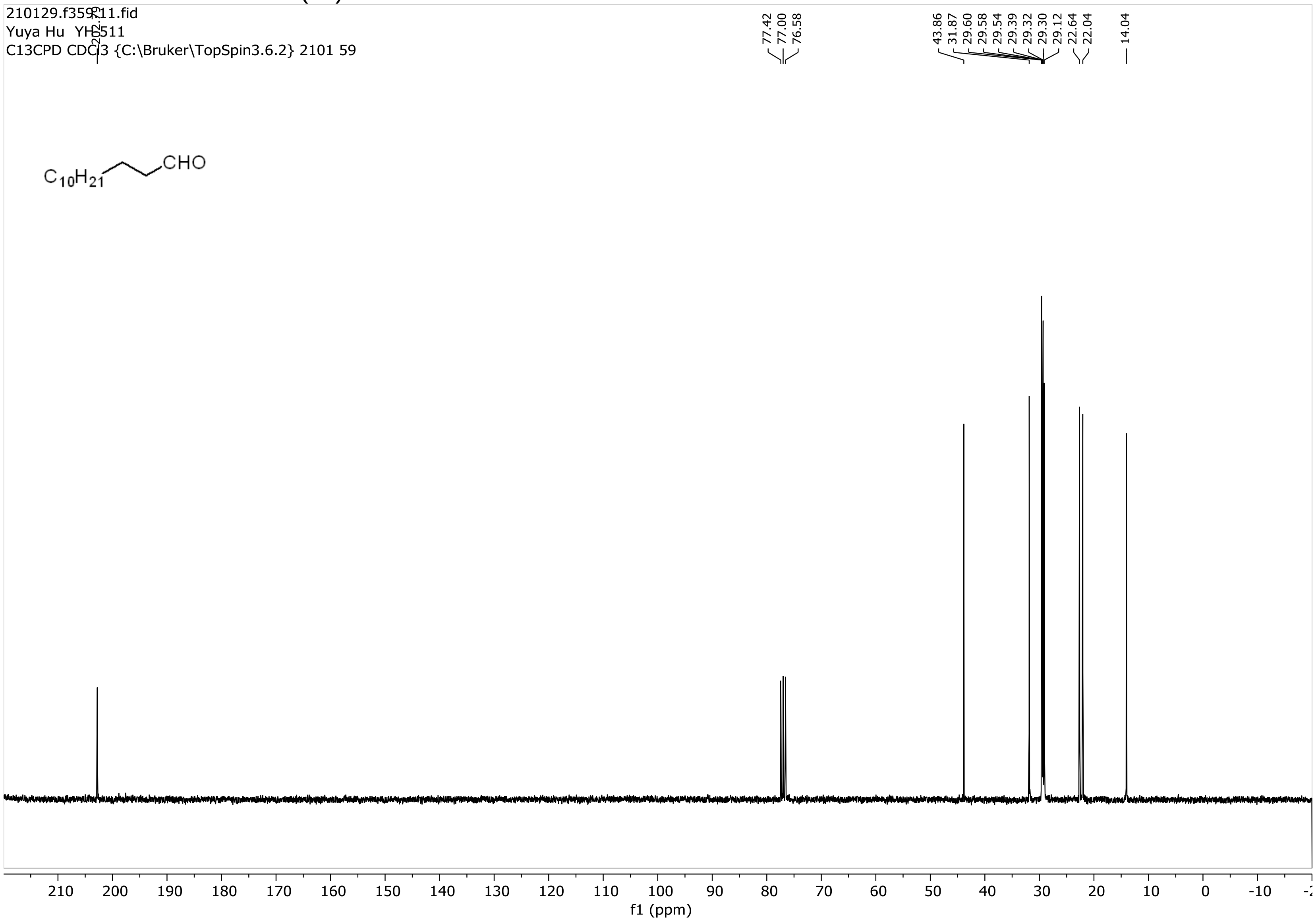

^{1}H NMR of 8-Hydroxy-4,8-dimethylnonanal (2d)

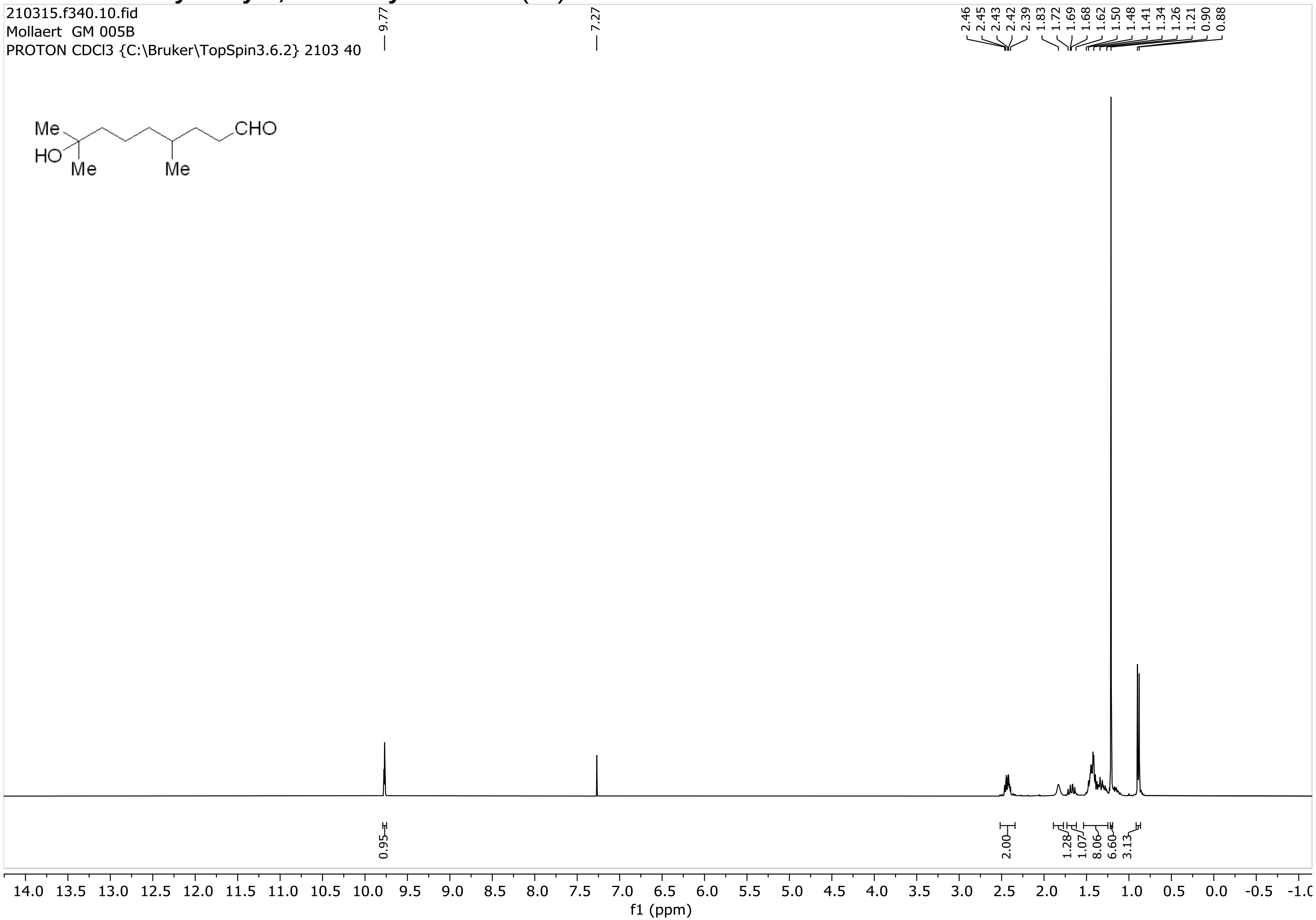

^{13}C NMR of 8-Hydroxy-4,8-dimethylnonanal (2d)

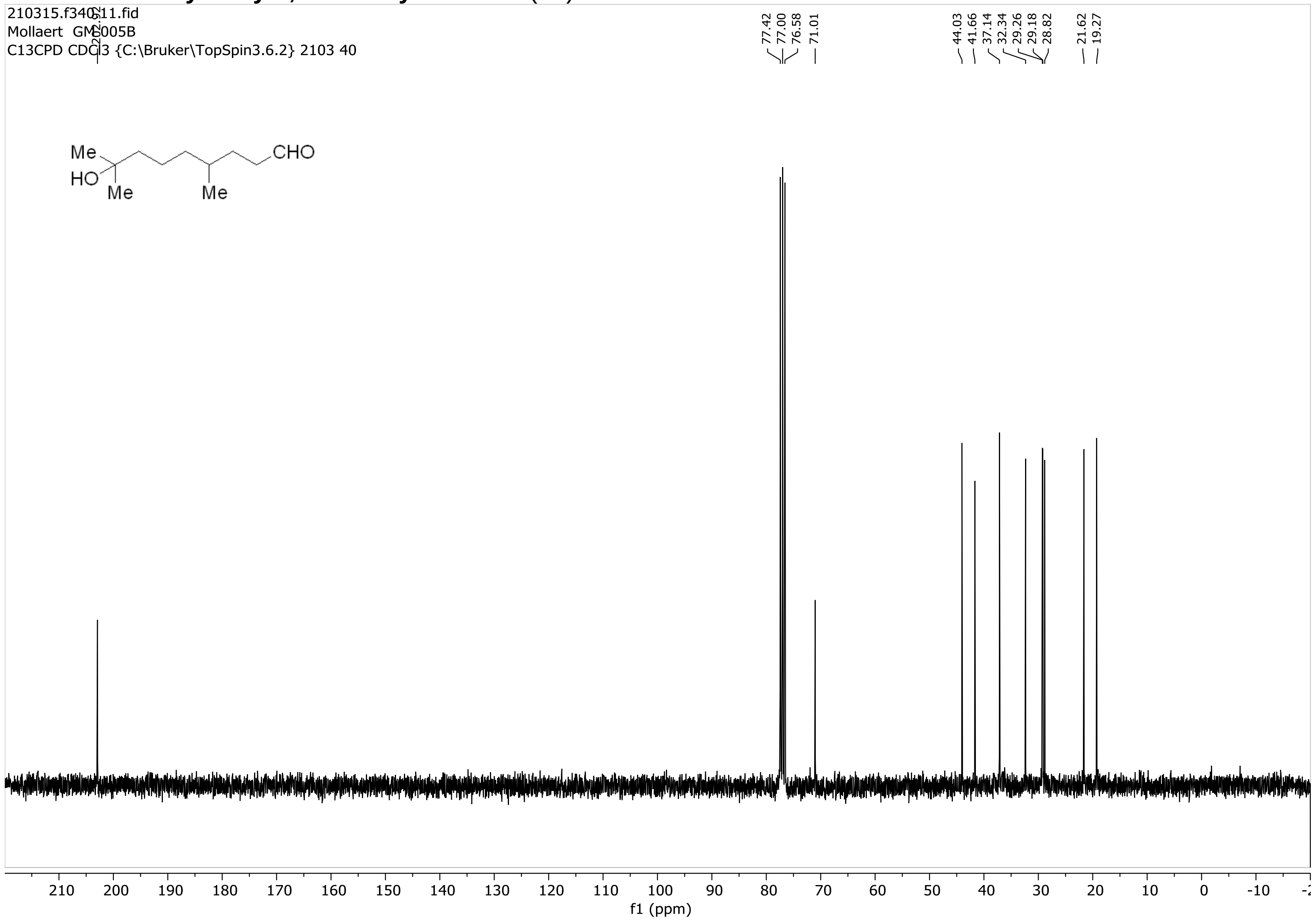

^{1}H NMR of 3-(Triethylsilyl)propanal (2e)

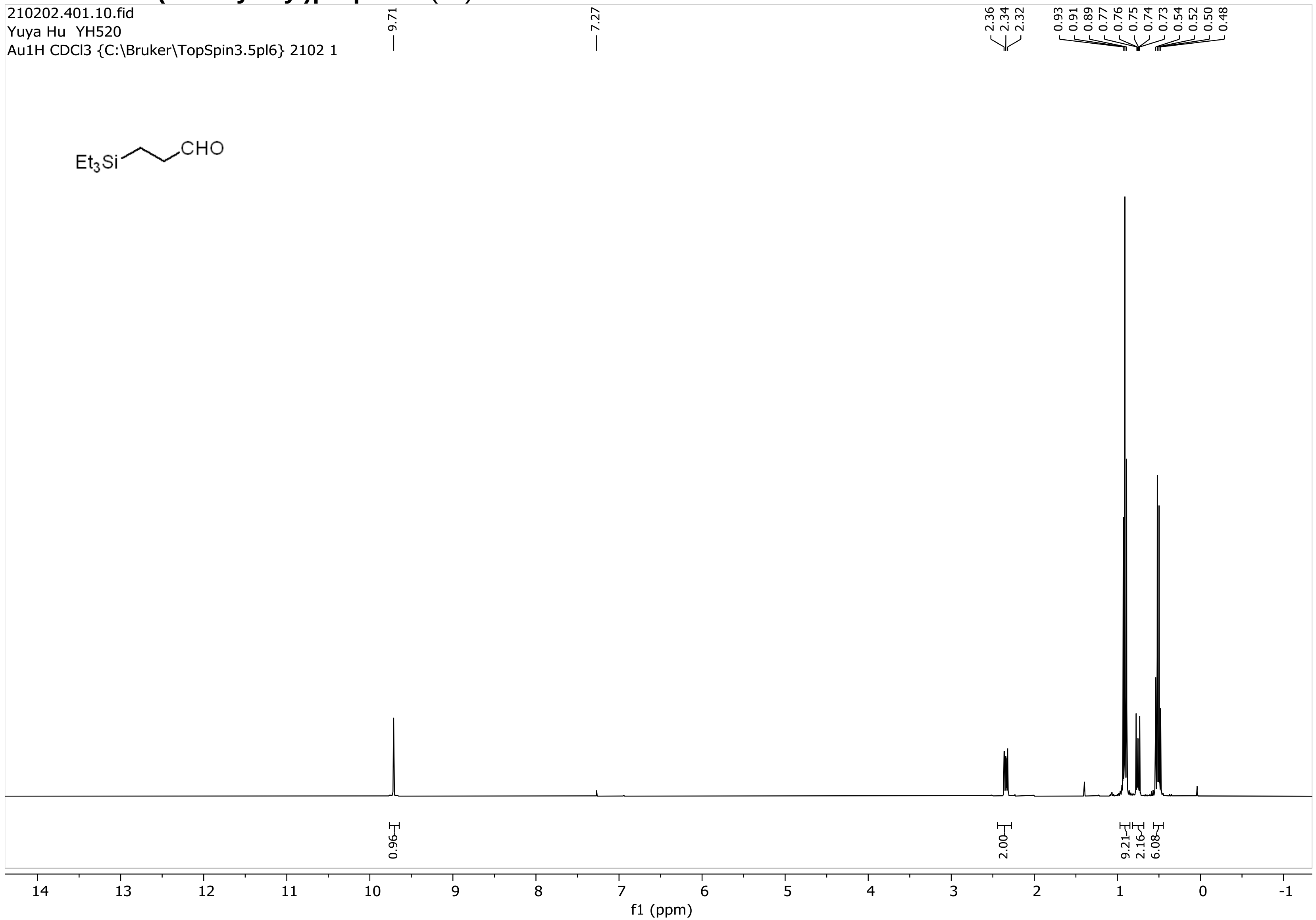

^{13}C NMR of 3-(Triethylsilyl)propanal (2e)

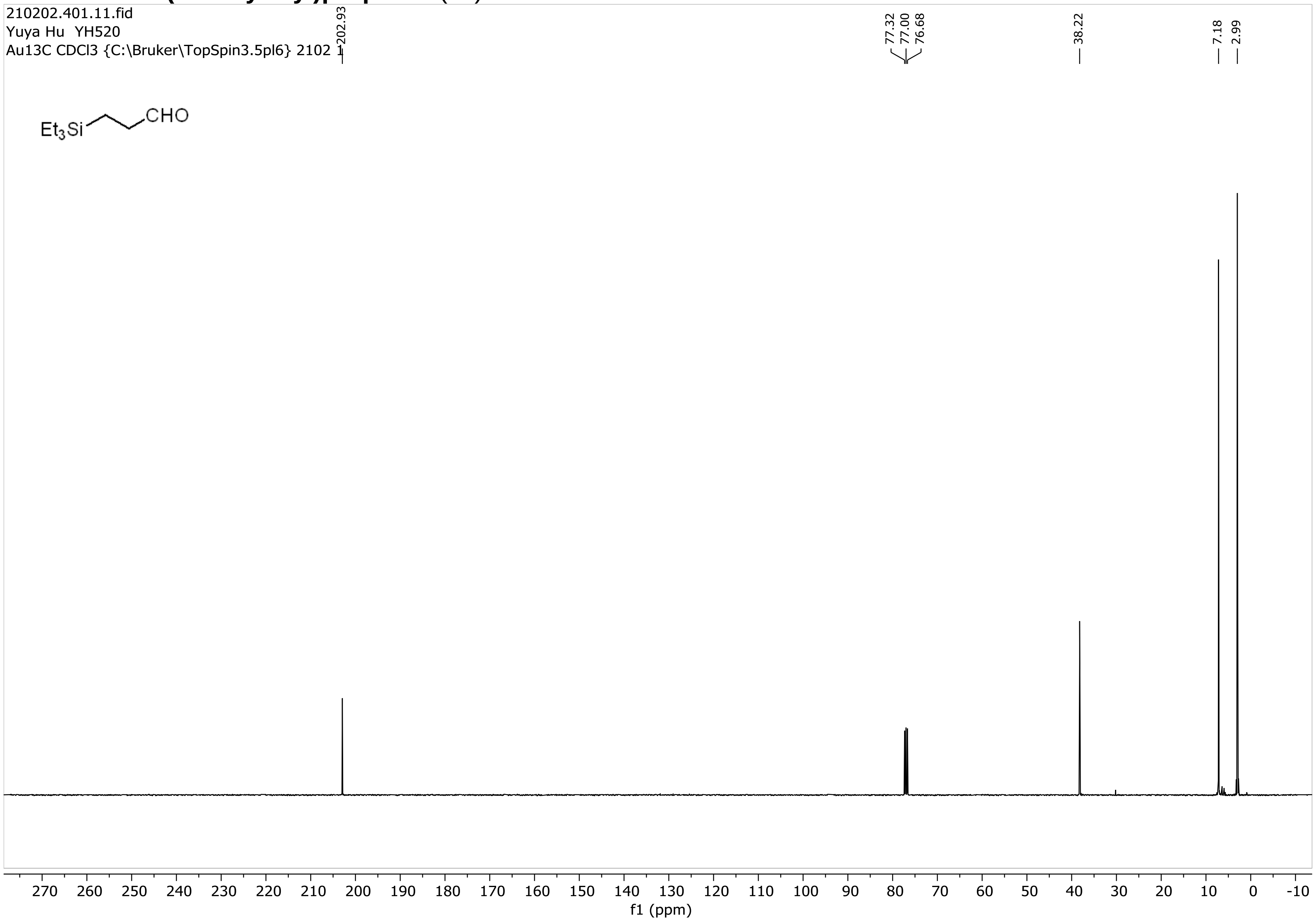

1H NMR of 4-Phenylbutanal (2f)

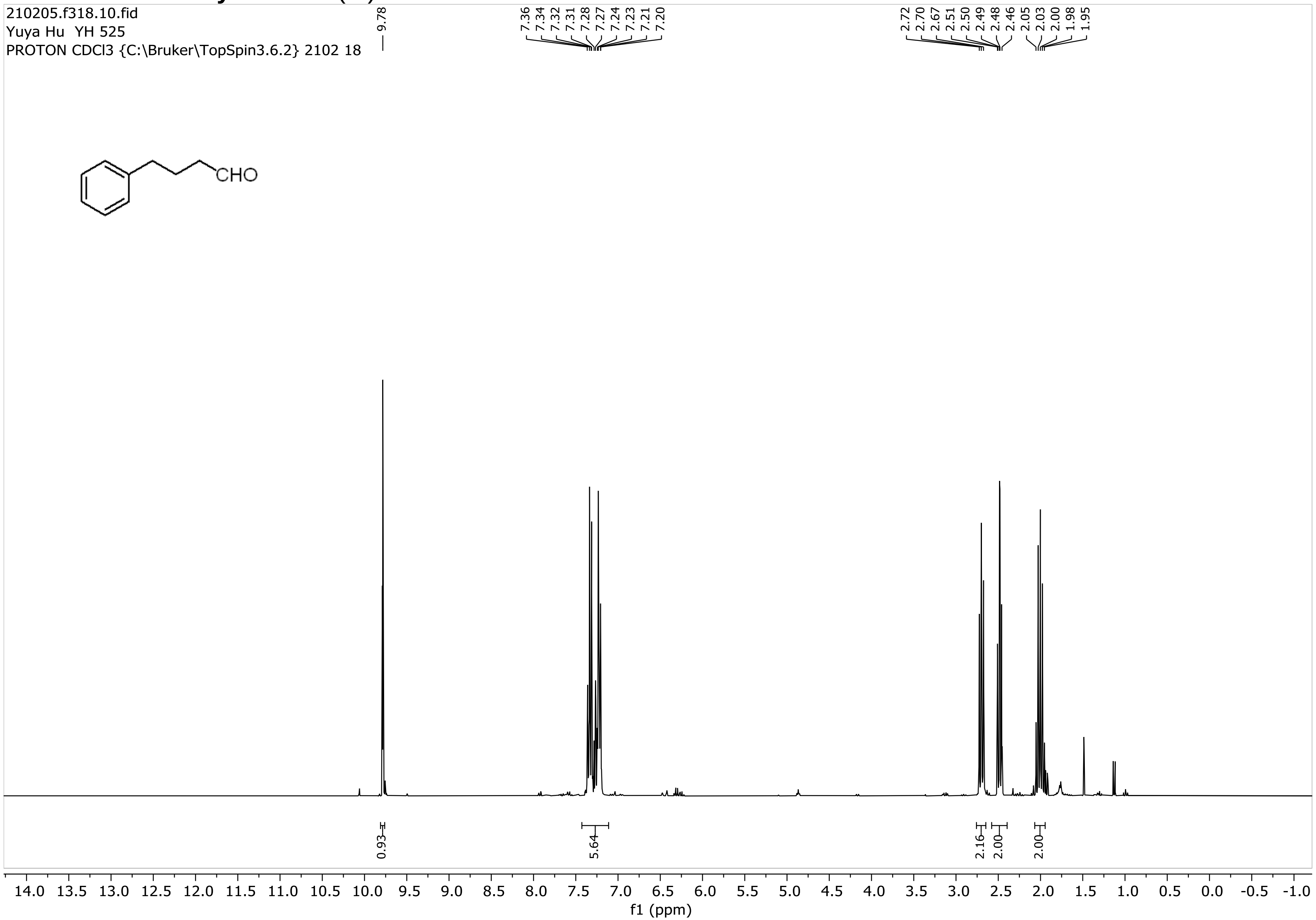

^{13}C NMR of 4-Phenylbutanal (2f)

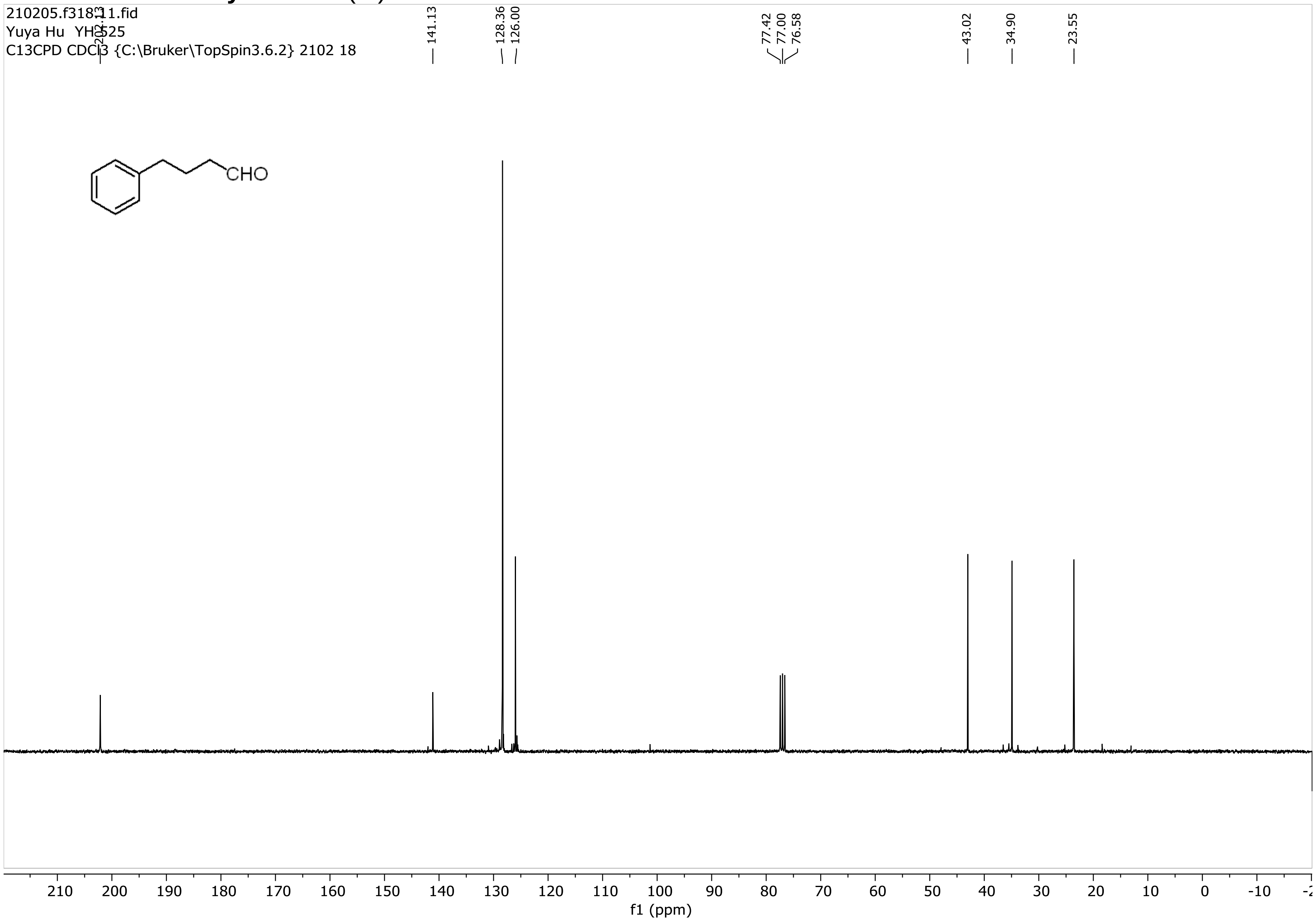

^{1}H NMR of 4-(4-Hydroxy-3-methoxyphenyl)butanal (2g)

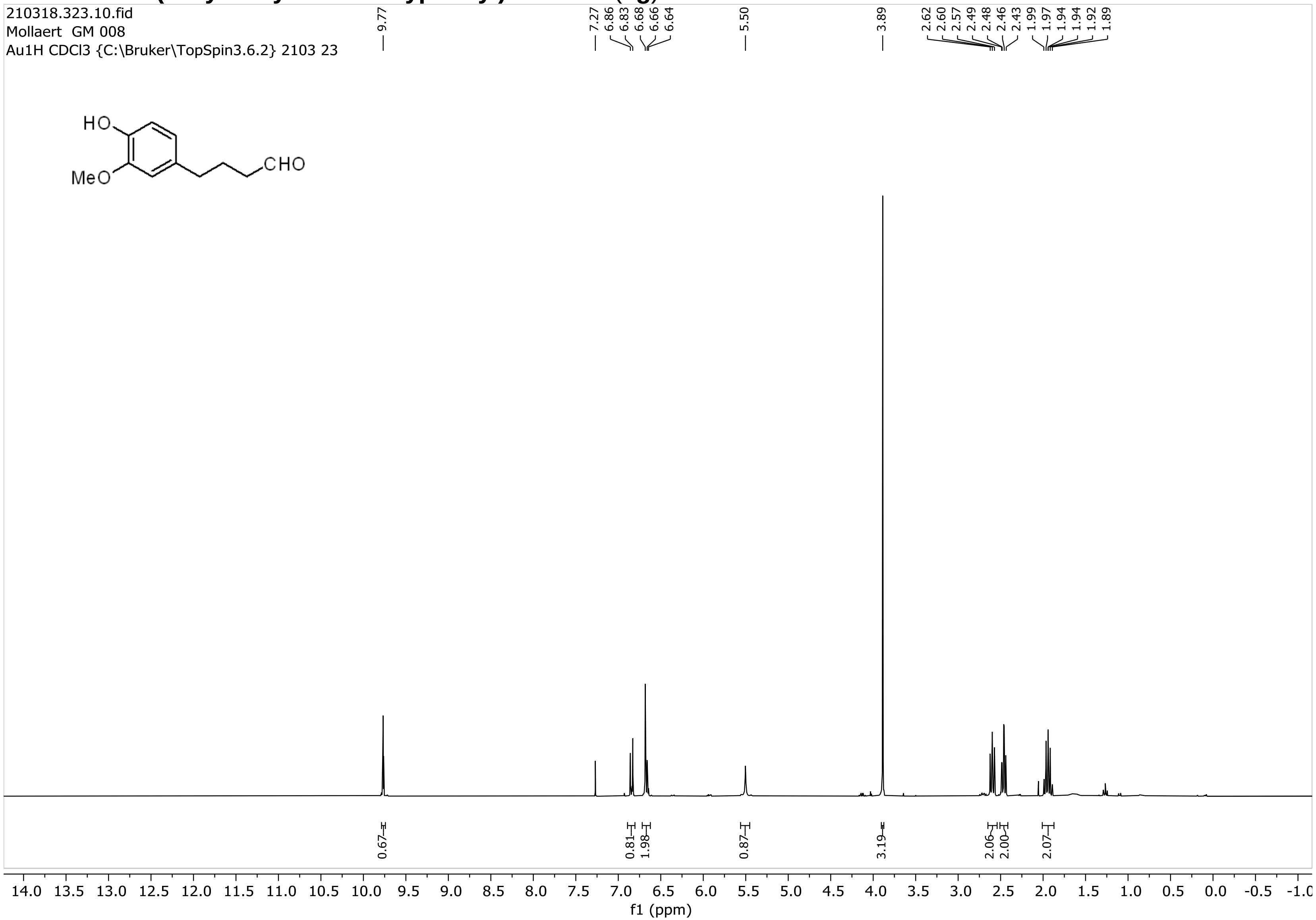

^{13}C NMR of 4-(4-Hydroxy-3-methoxyphenyl)butanal (2g)

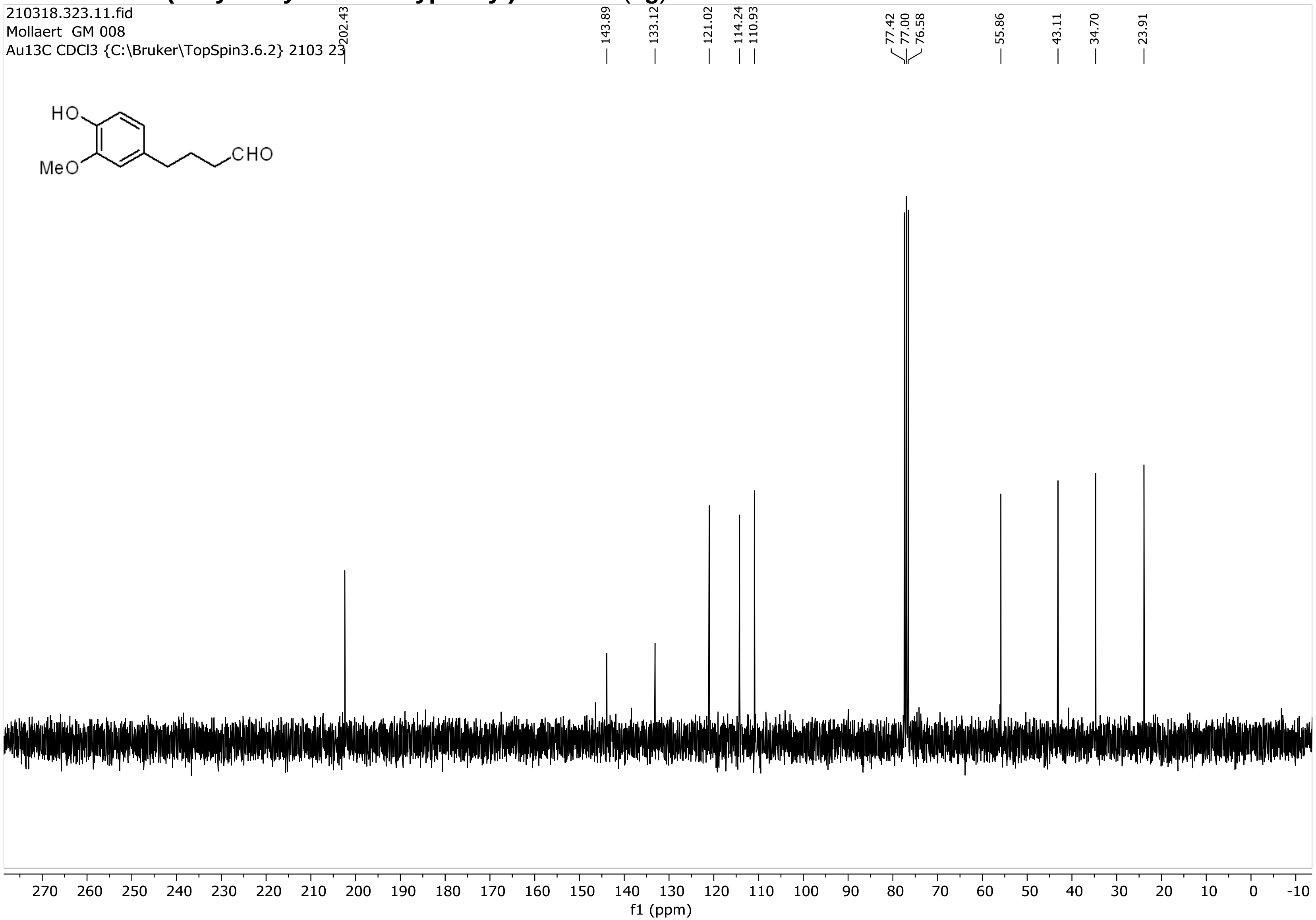

^{1}H NMR of 2-Phenylpropanal (2h)

220419.419.10.fid
Rui Sang sr-5165-2
Au1H CDCl3 {C:\Bruker\TopSpin3.5pl6} 2204 19

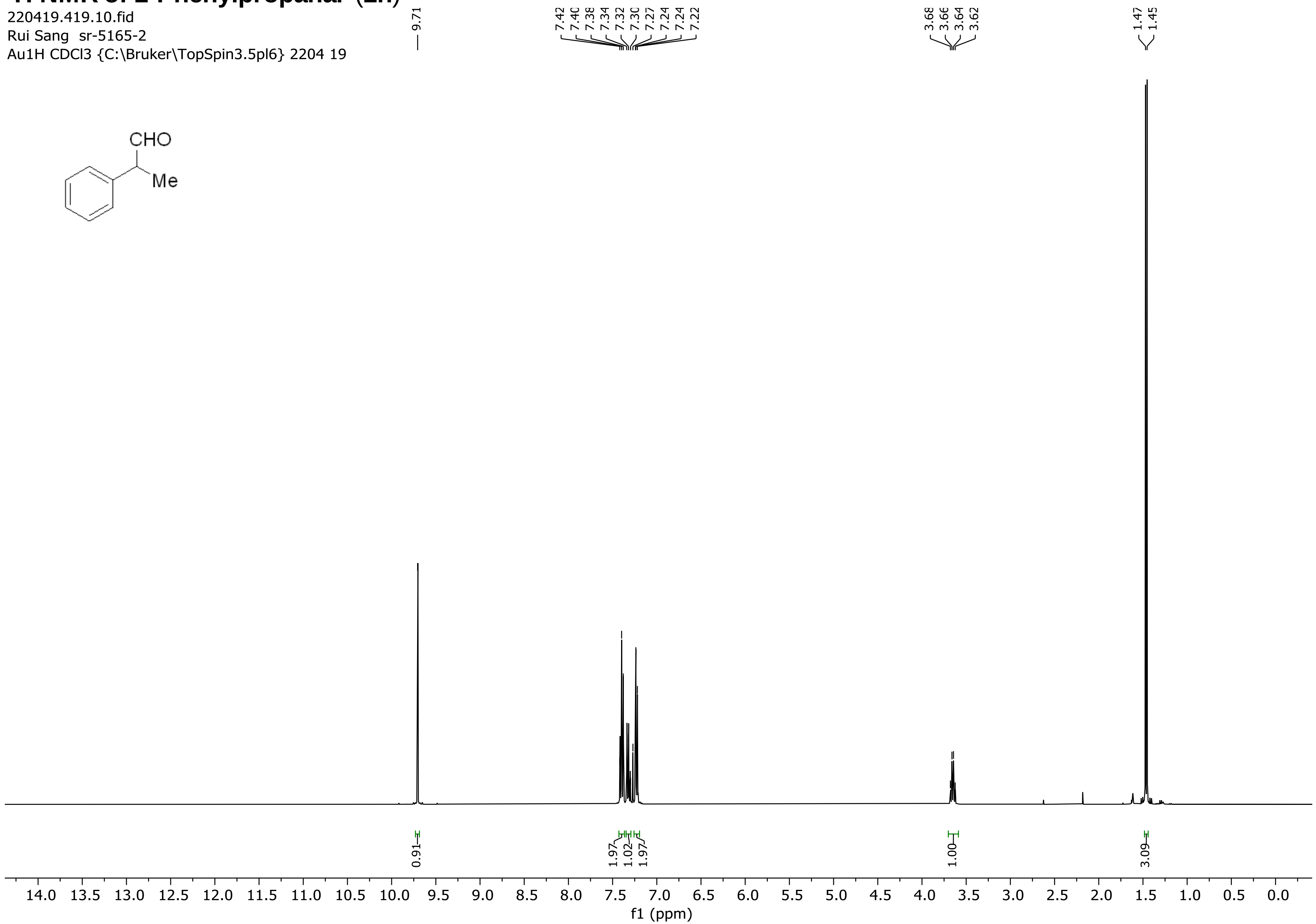

^{13}C NMR of 2-Phenylpropanal (2h)

220419.419.11.fid
Rui Sang sr-5165-2
Au13C CDCl3 {C:\Bruker\TopSpin3.5pl6} 2204 19

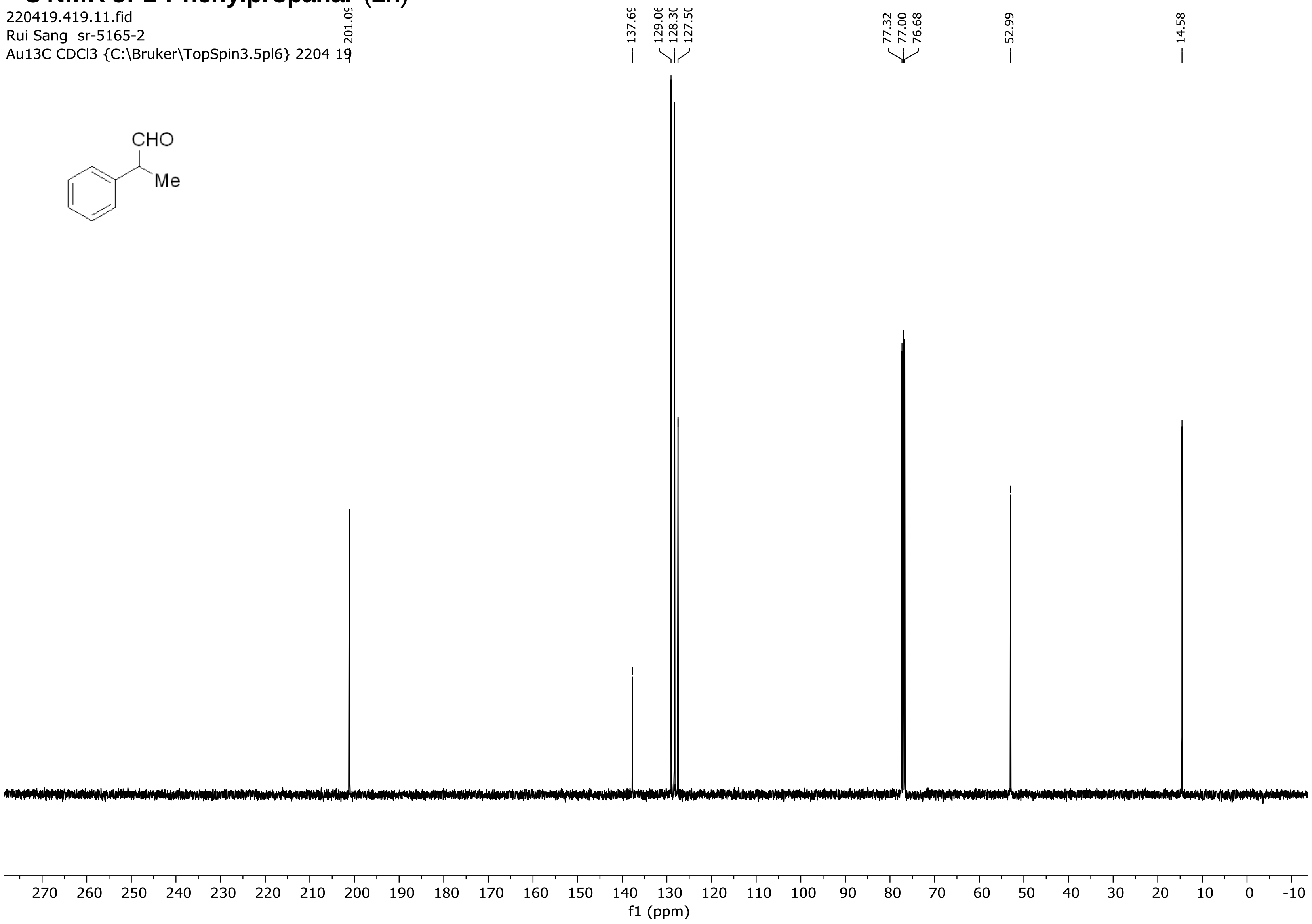

^{1}H NMR of 2-(*p*-Tolyl)propanal (2i)

220419.423.10.fid
Rui Sang sr-5159-2
Au1H CDCl3 {C:\Bruker\TopSpin3.5pl6} 2204 23

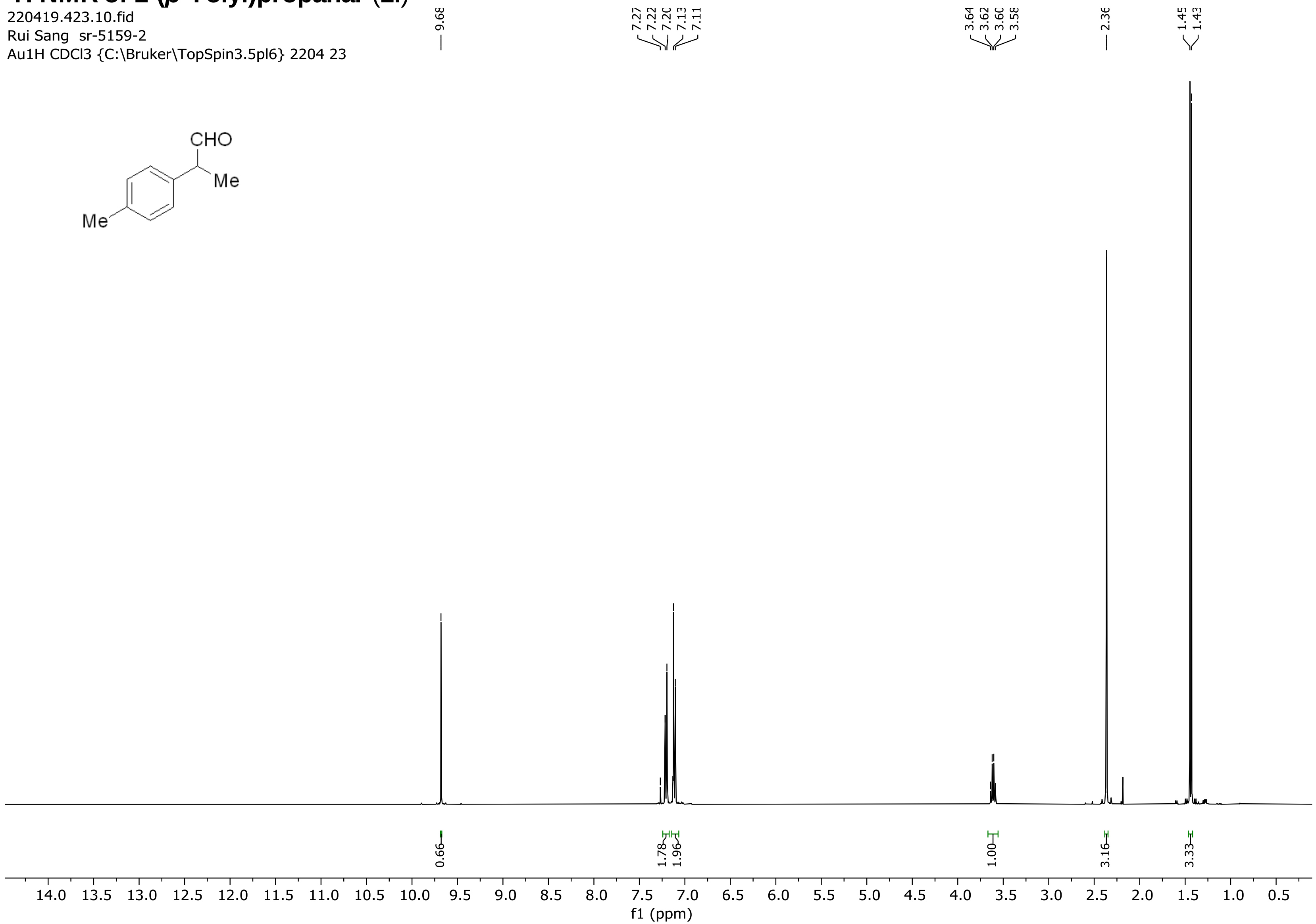

^{13}C NMR of 2-(*p*-Tolyl)propanal (2i)

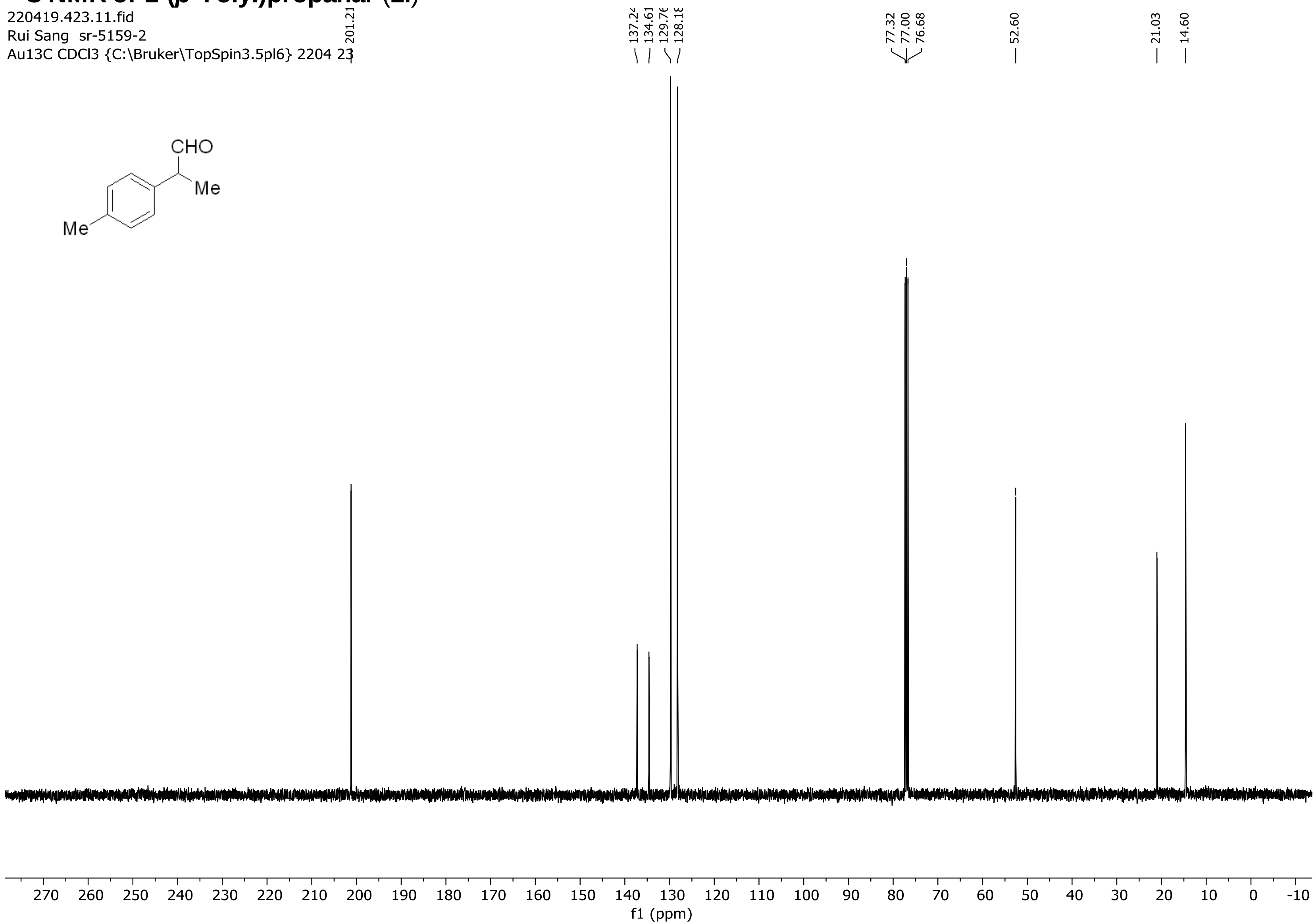

^{1}H NMR of 2-(4-Bromophenyl)propanal (2j)

220419.421.10.fid
Rui Sang sr-5166-2
Au1H CDCl3 {C:\Bruker\TopSpin3.5pl6} 2204 21

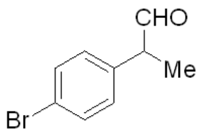

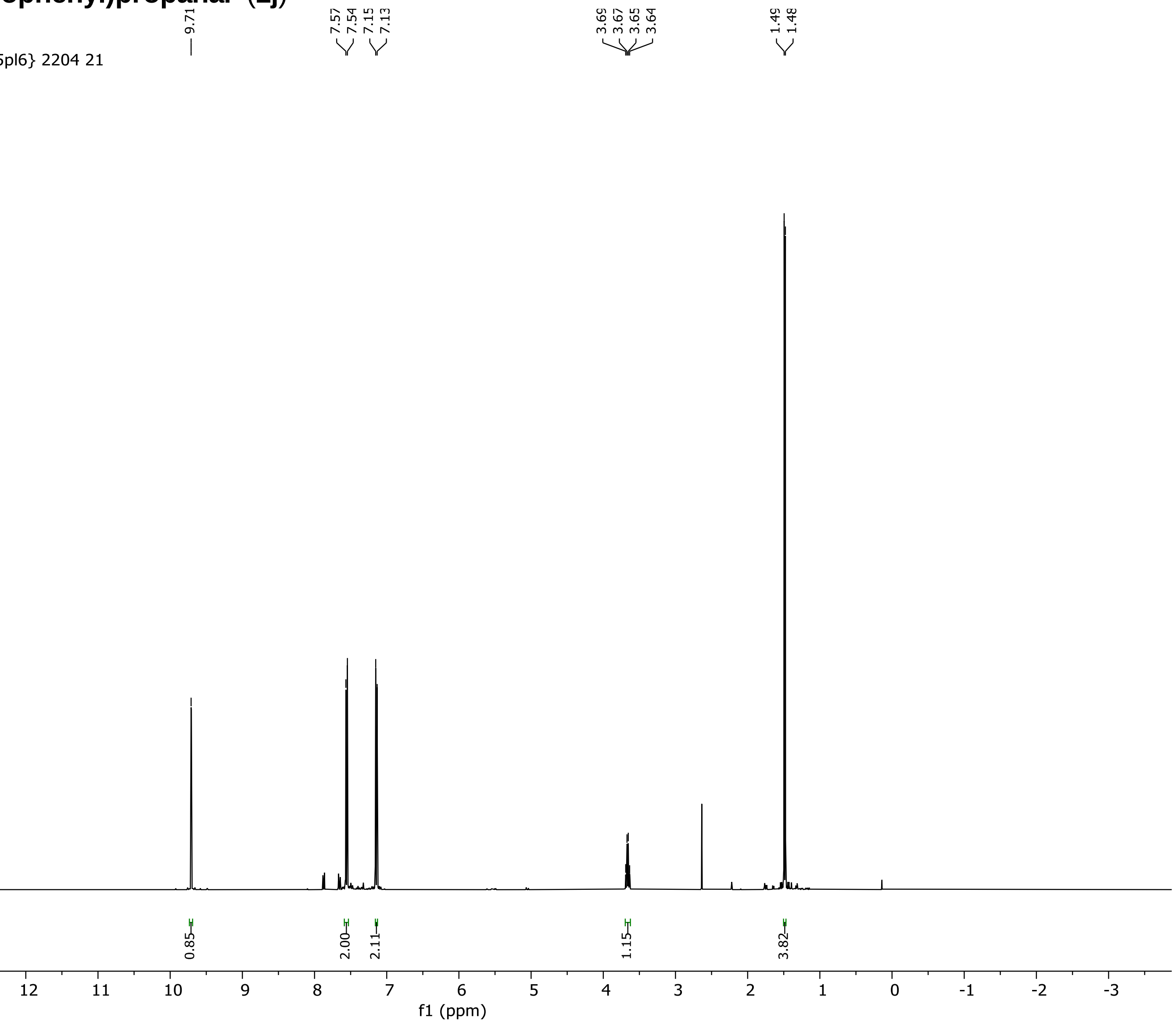

^{13}C NMR of 2-(4-Bromophenyl)propanal (2j)

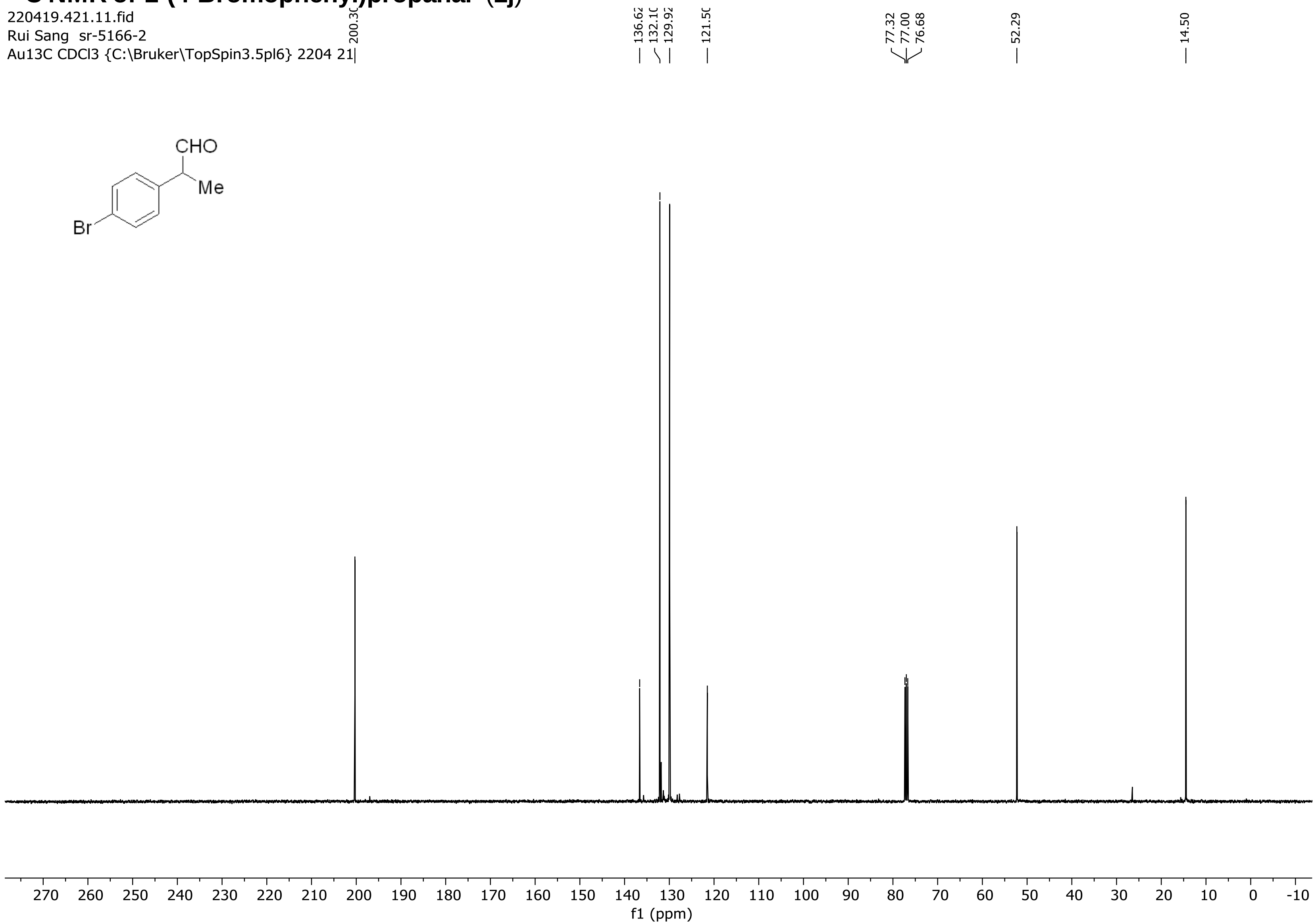

^{1}H NMR of Methyl 2-phenylacrylate (4a)

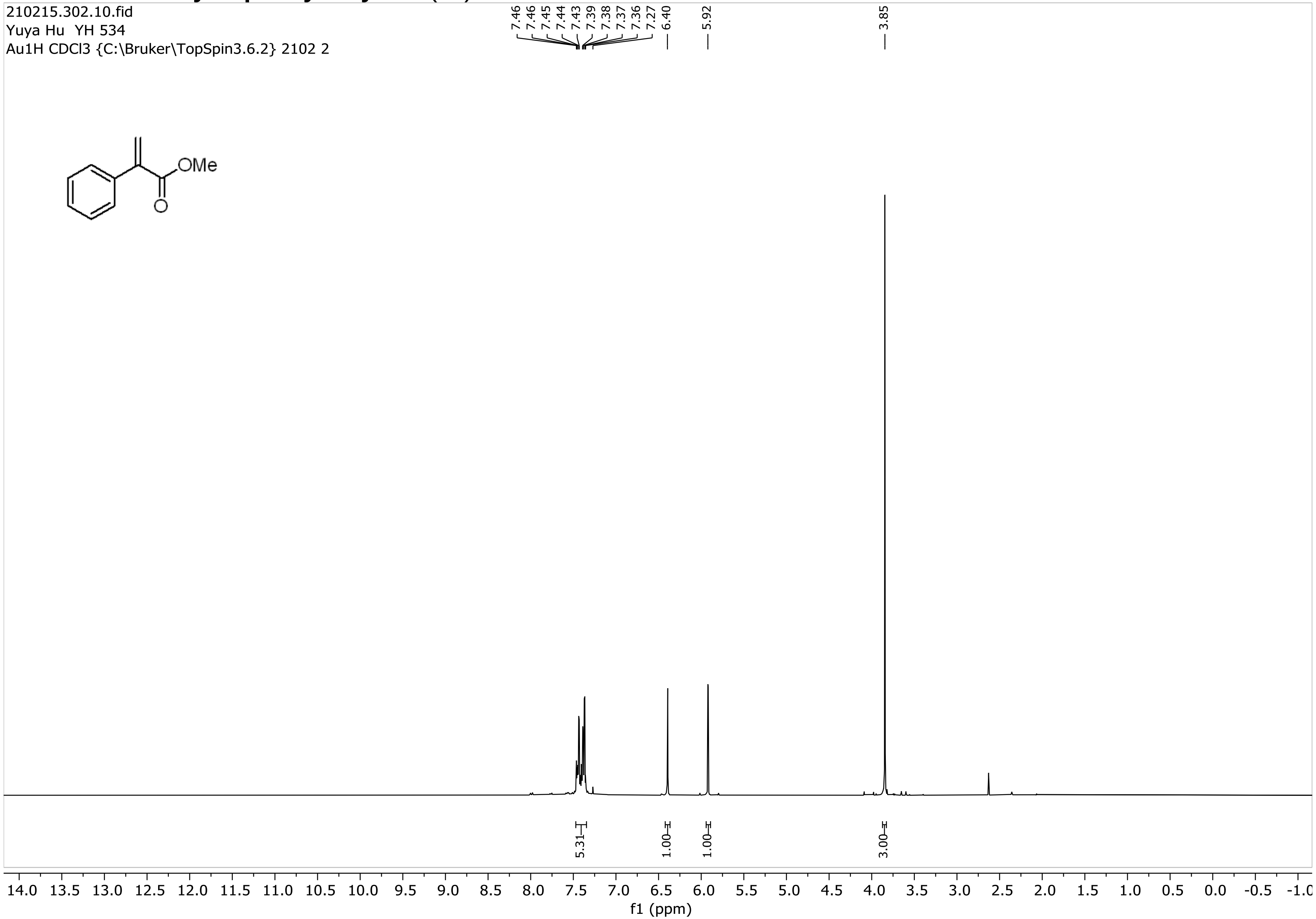

^{13}C NMR of Methyl 2-phenylacrylate (4a)

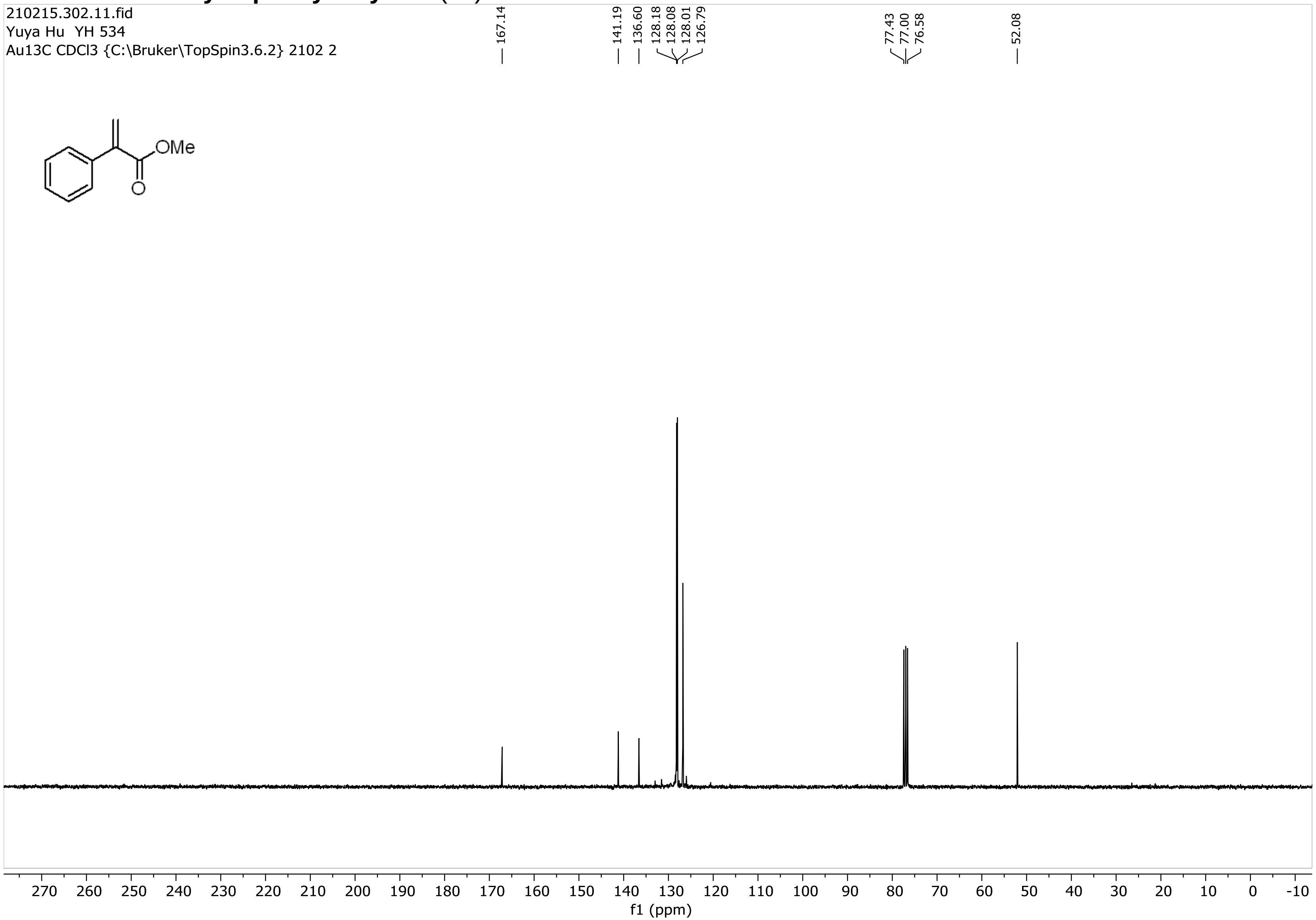

^{1}H NMR of Methyl 2-(4-methoxy-2-methylphenyl)acrylate (4b)

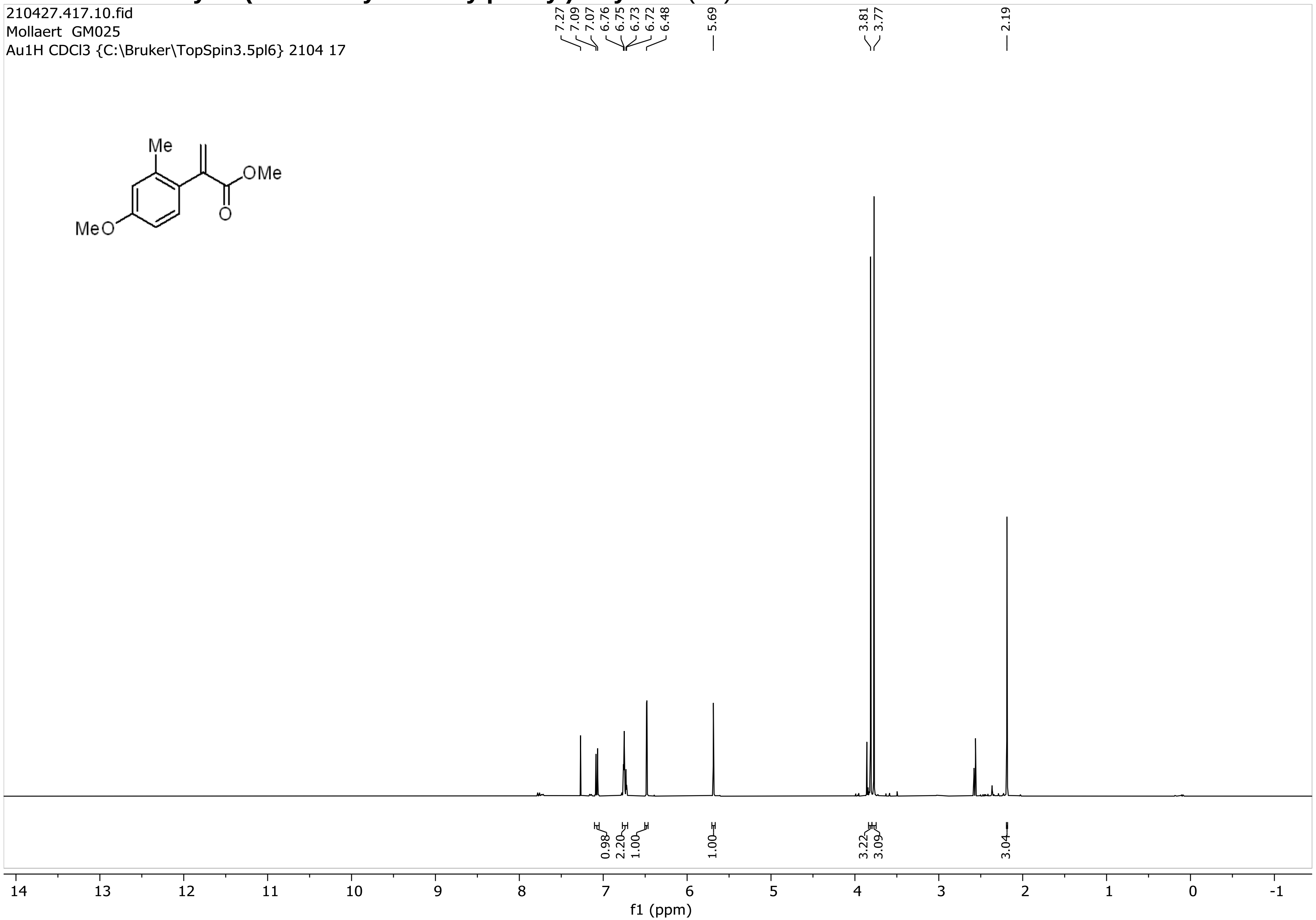

^{13}C NMR of Methyl 2-(4-methoxy-2-methylphenyl)acrylate (4b)

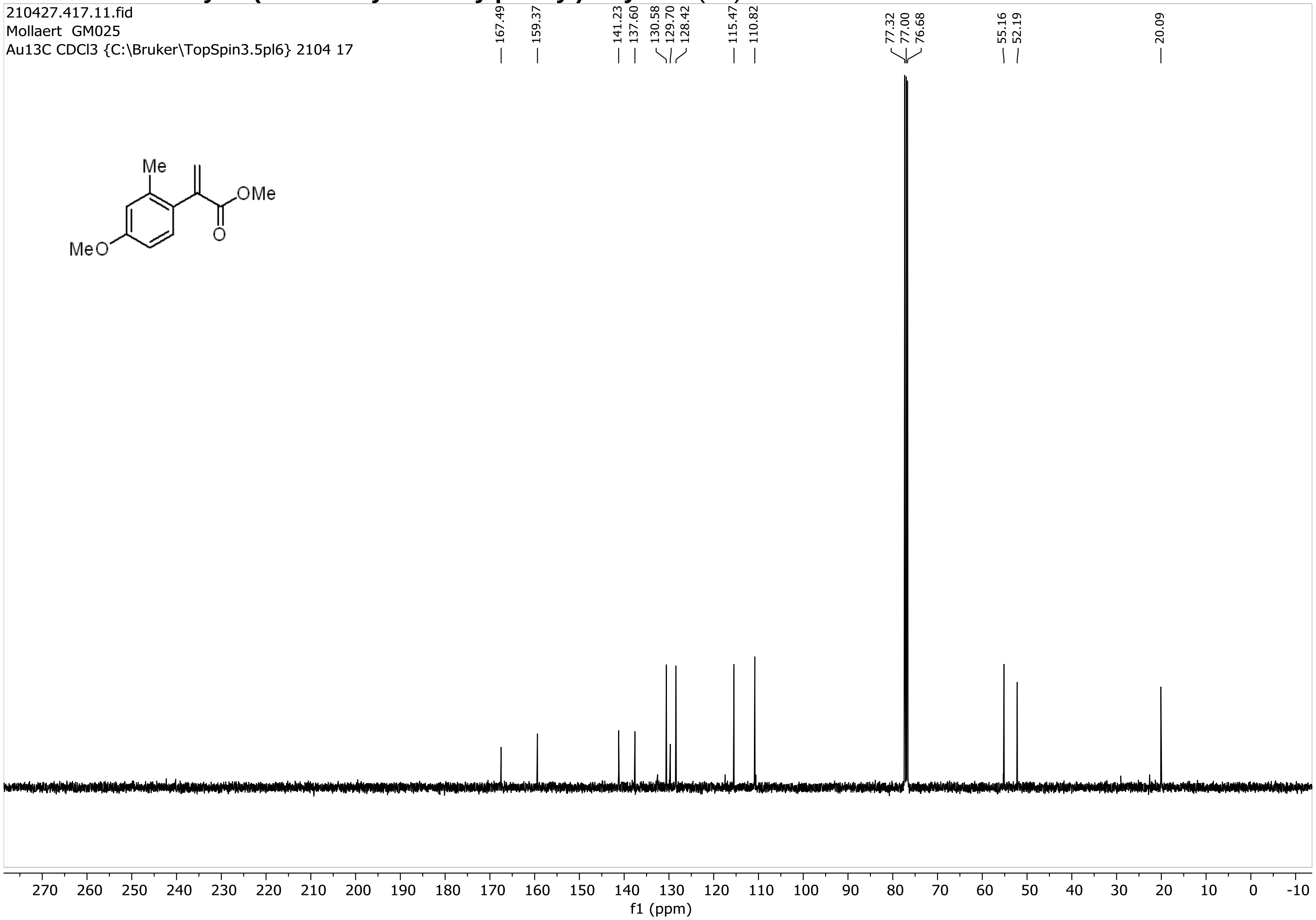

^{1}H NMR of Methyl 5-chloro-2-methylenepentanoate (4c)

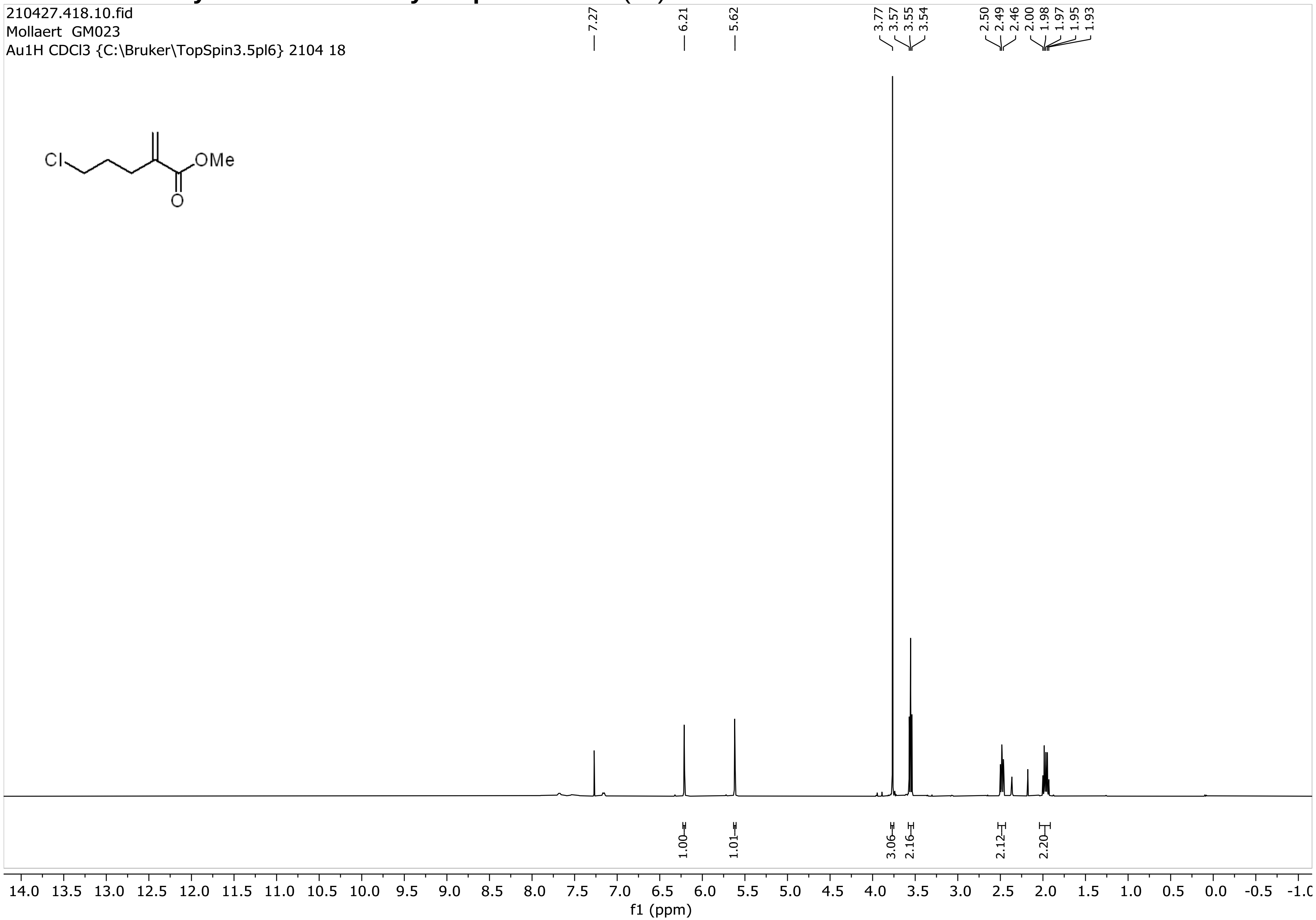

^{13}C NMR of Methyl 5-chloro-2-methylenepentanoate (4c)

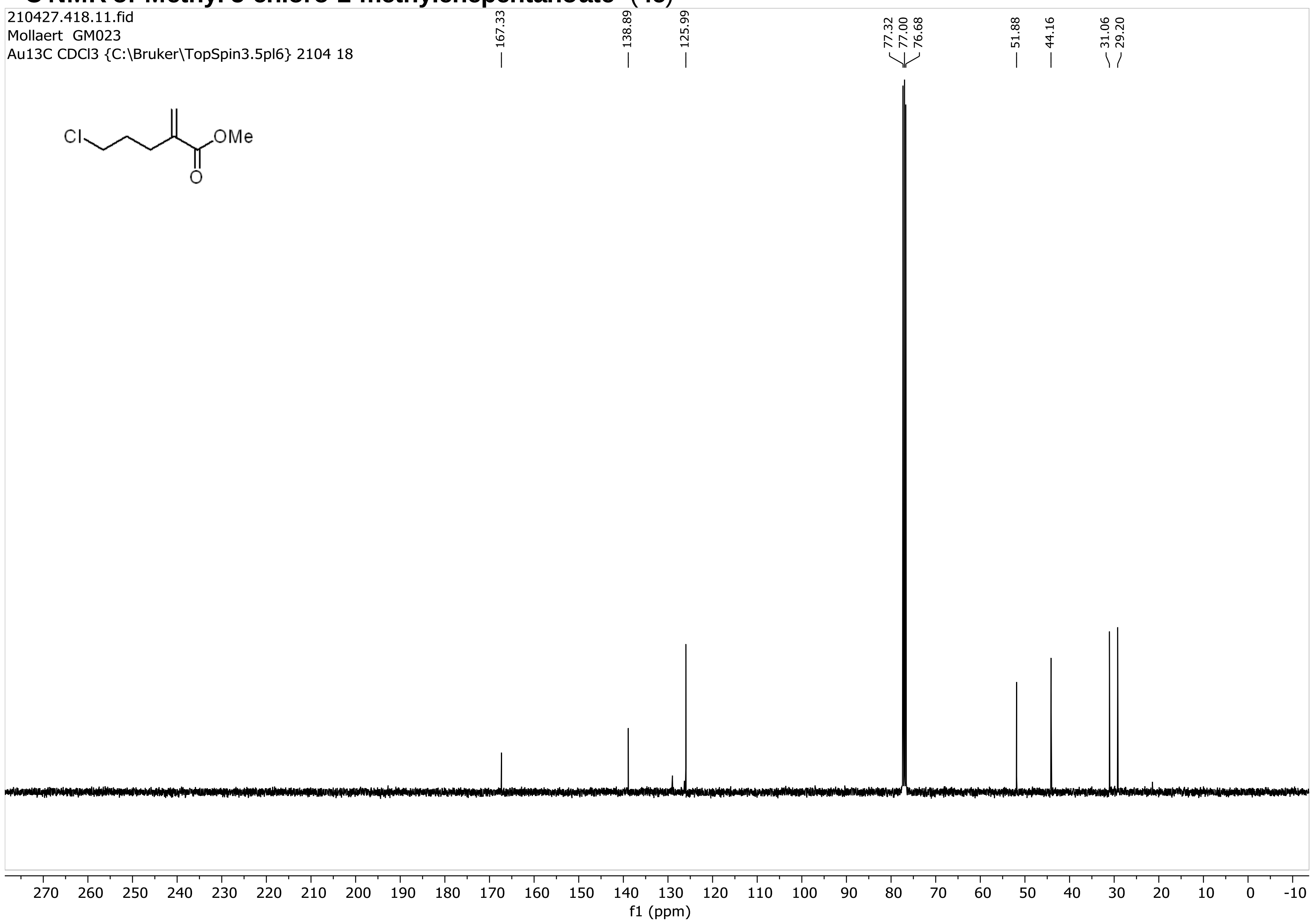

^{1}H NMR of Methyl 5-cyano-2-methylenepentanoate (4d)

210331.f362.10.fid
Mollaert GM 15
PROTON CDCl3 {C:\Bruker\TopSpin3.6.2} 2103 2

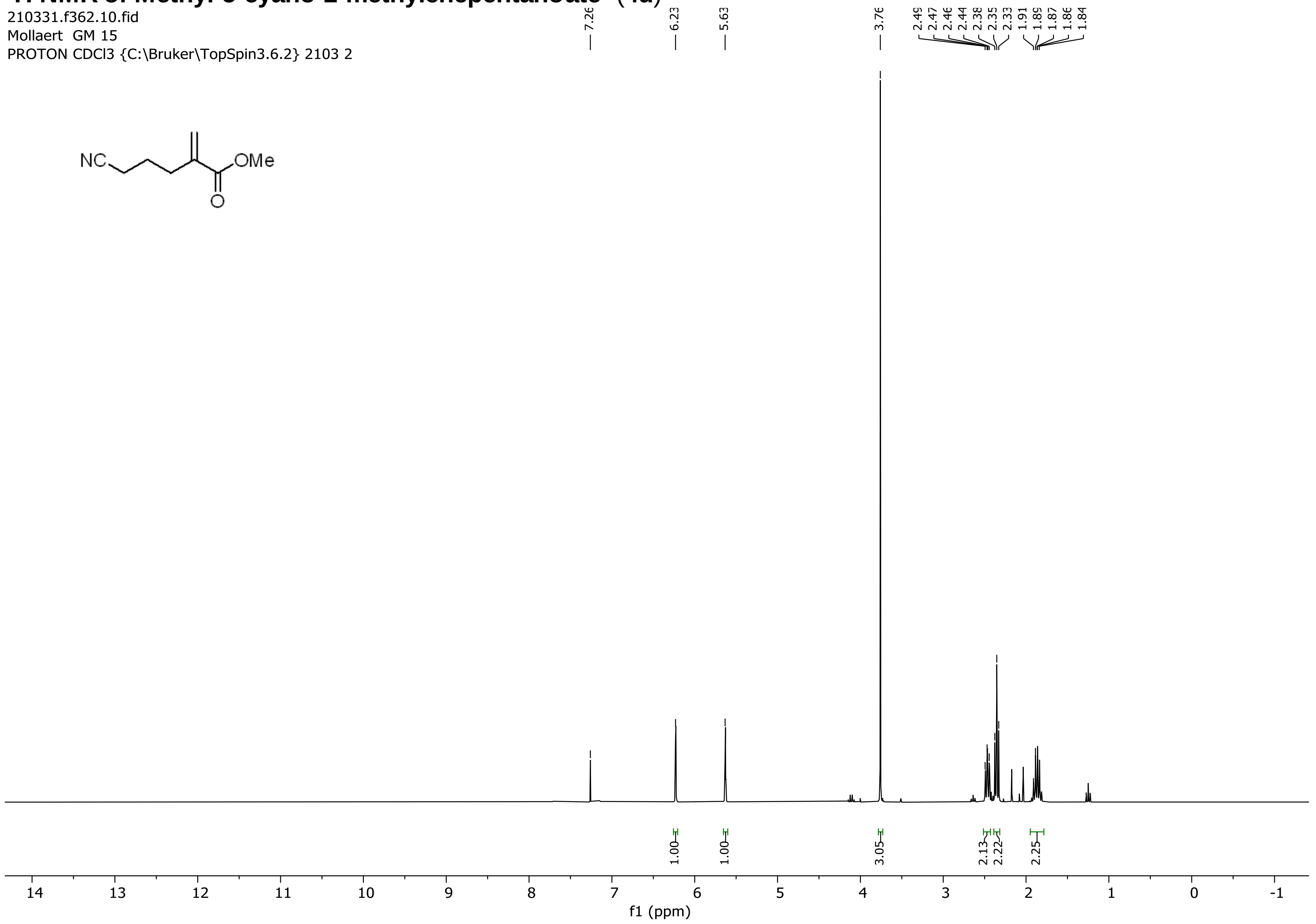

^{13}C NMR of Methyl 5-cyano-2-methylenepentanoate (4d)

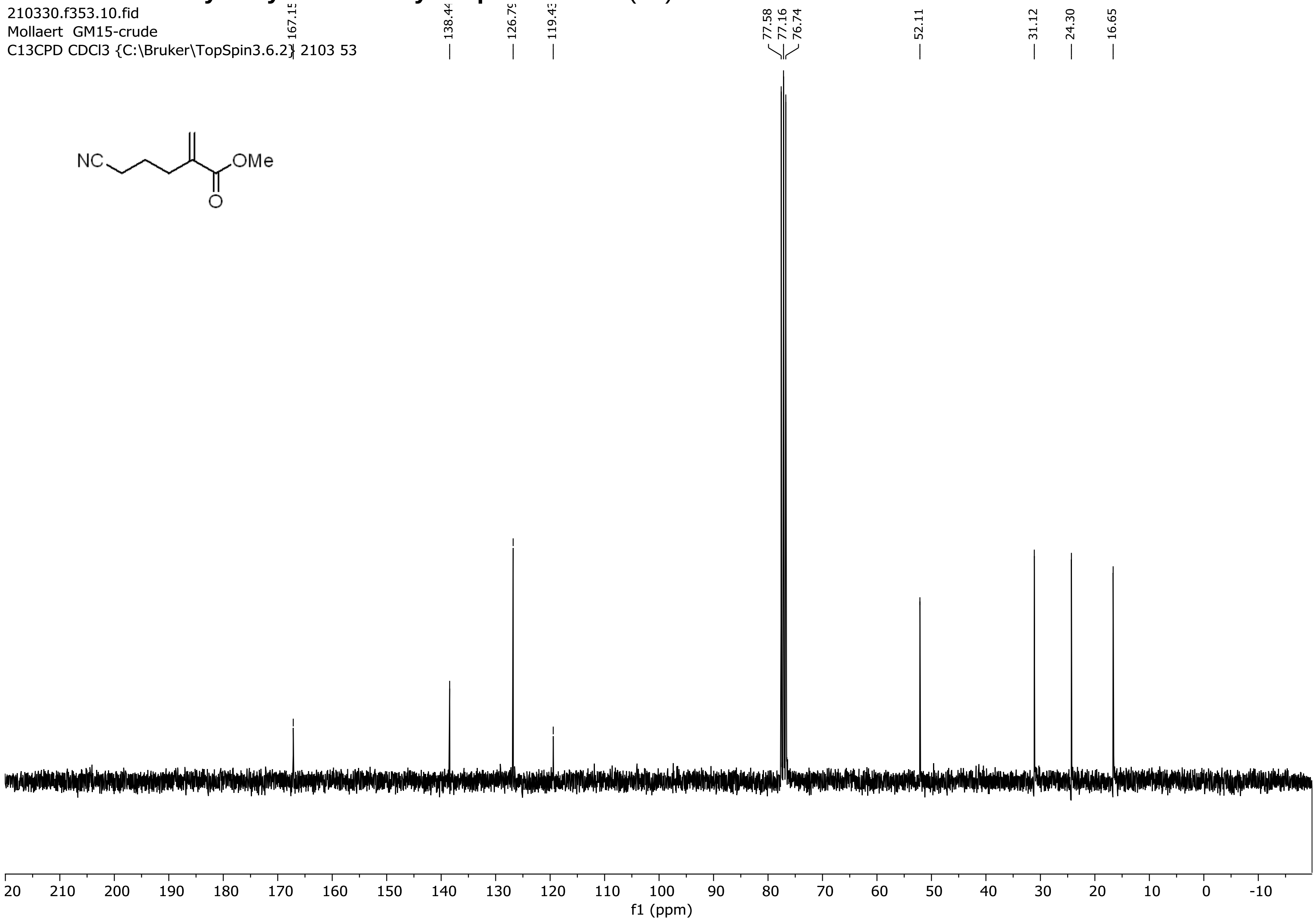

^{1}H NMR of (4-Methoxyphenyl)(piperidin-1-yl)methanone (7a)

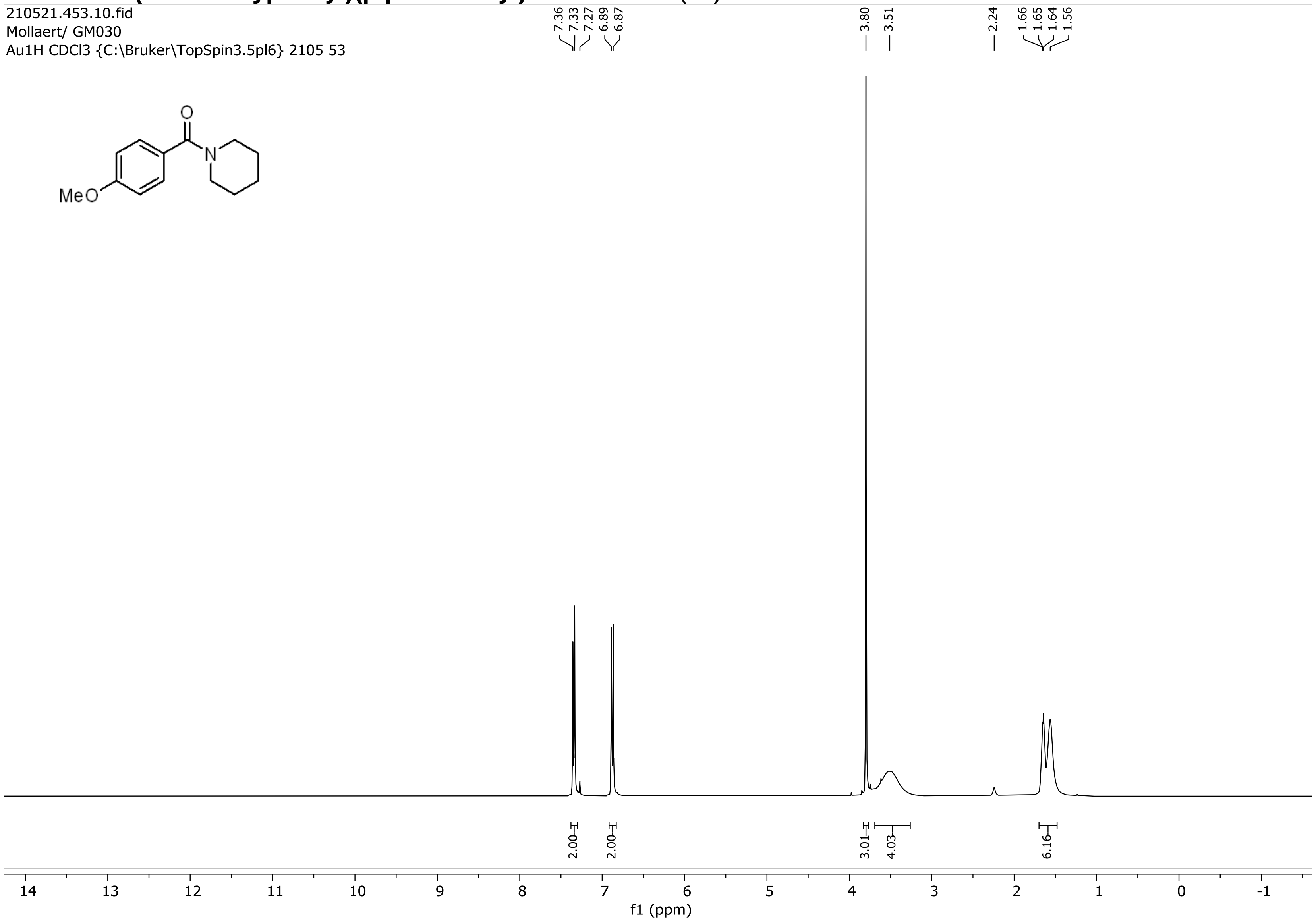

13C NMR of (4-Methoxyphenyl)(piperidin-1-yl)methanone (7a)

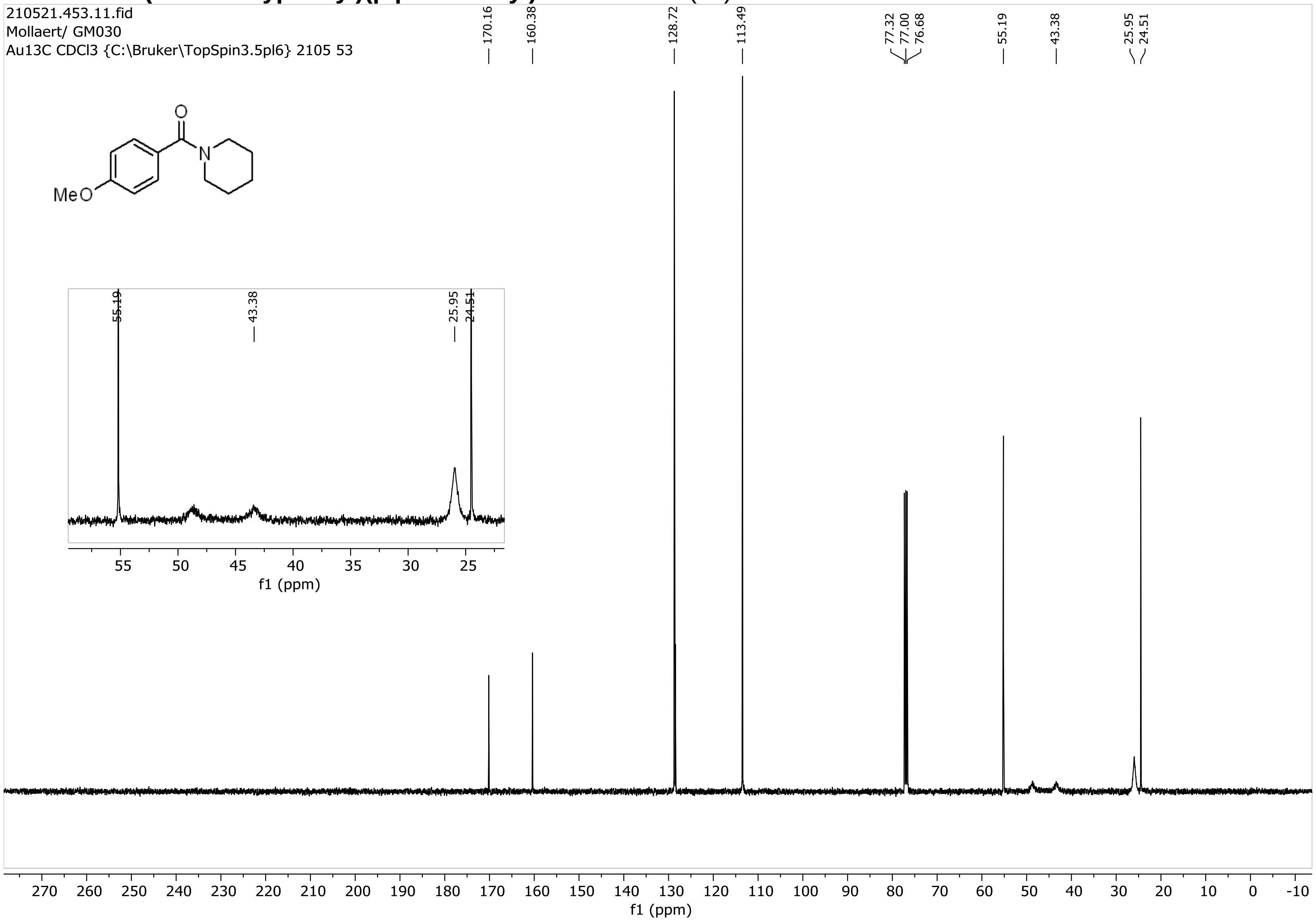

^{1}H NMR of 1-(4-(Piperidine-1-carbonyl)phenyl)ethan-1-one (7b)

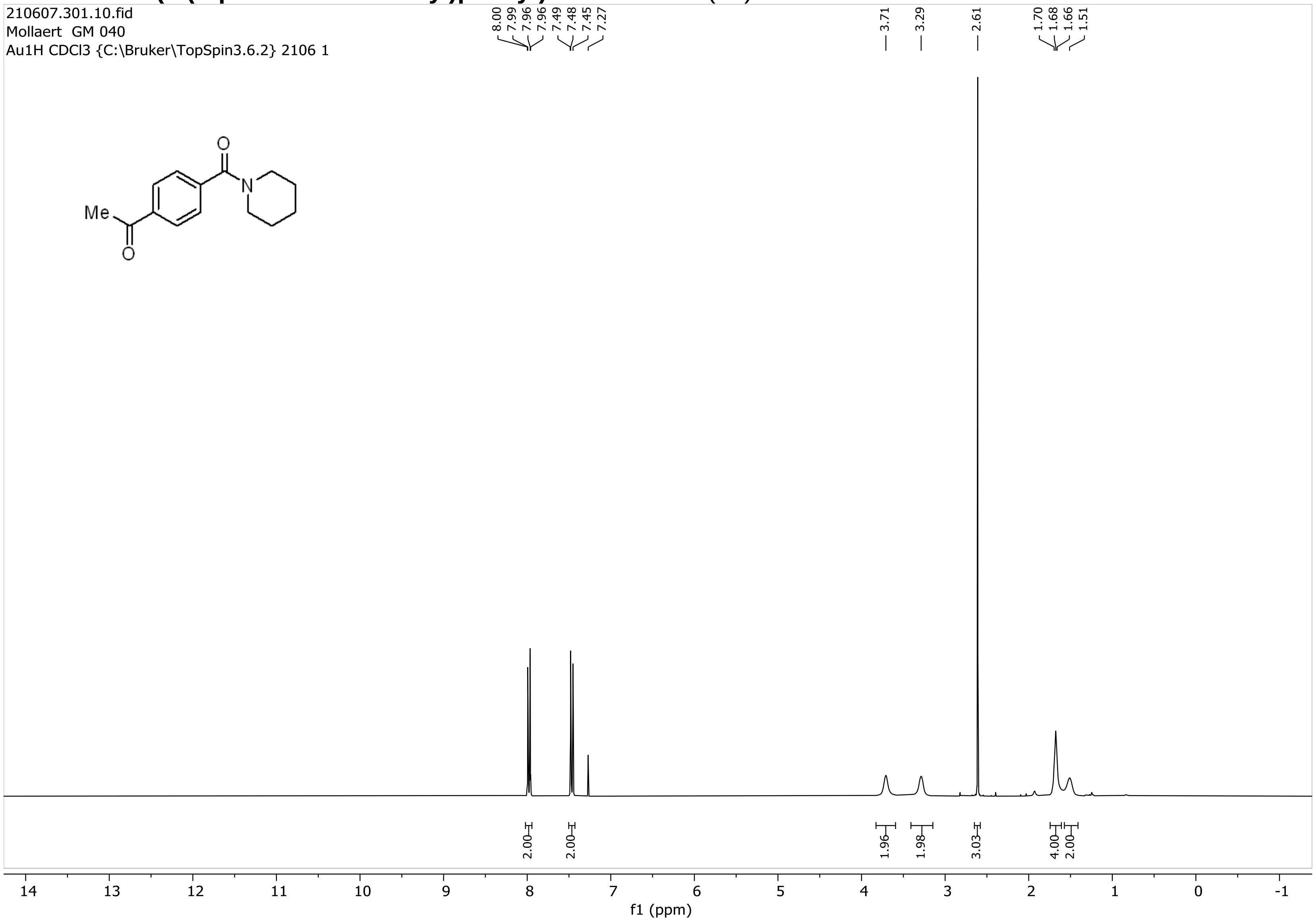

^{13}C NMR of 1-(4-(Piperidine-1-carbonyl)phenyl)ethan-1-one (7b)

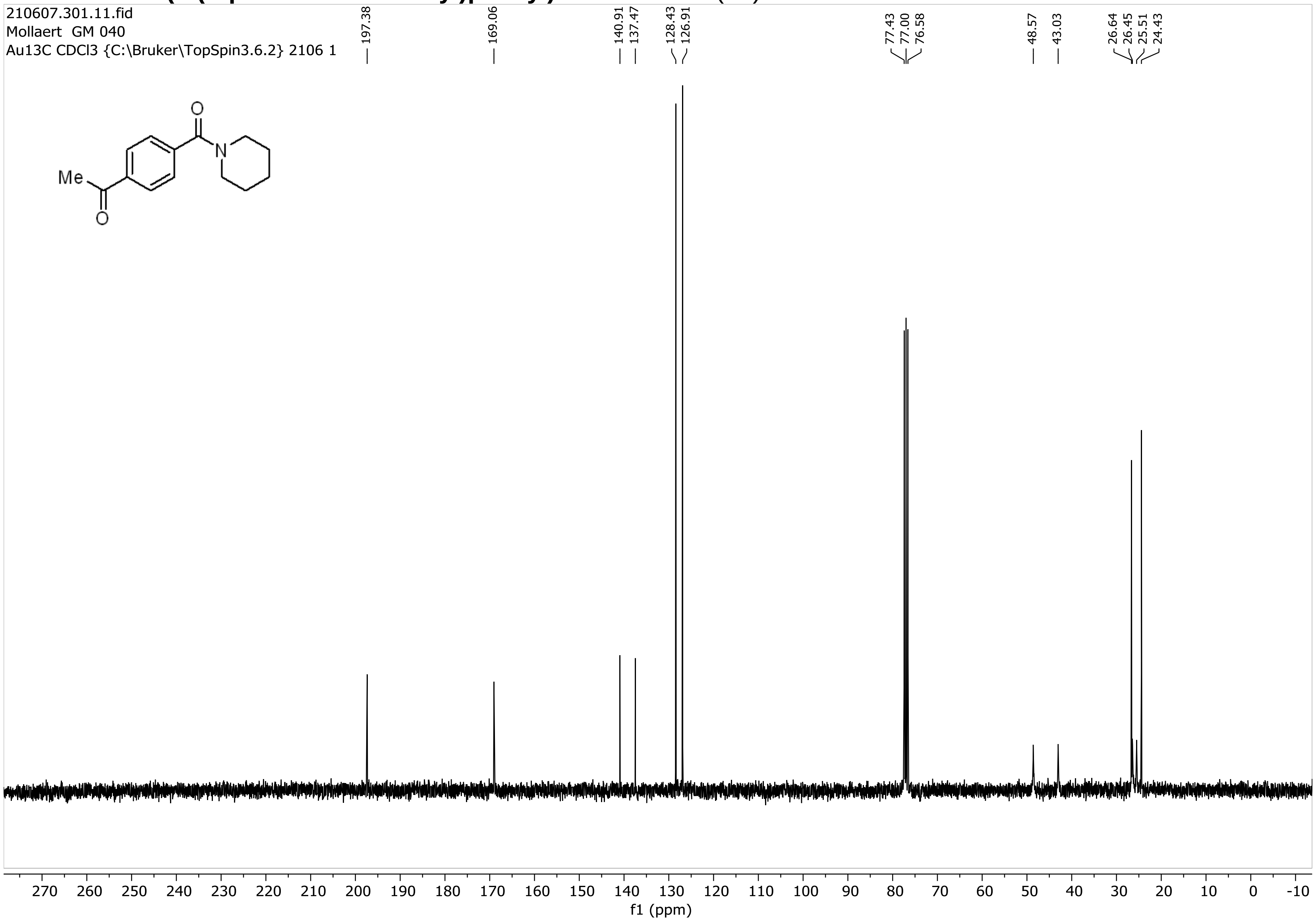

^{1}H NMR of (4-Methoxyphenyl)(morpholino)methanone (7c)

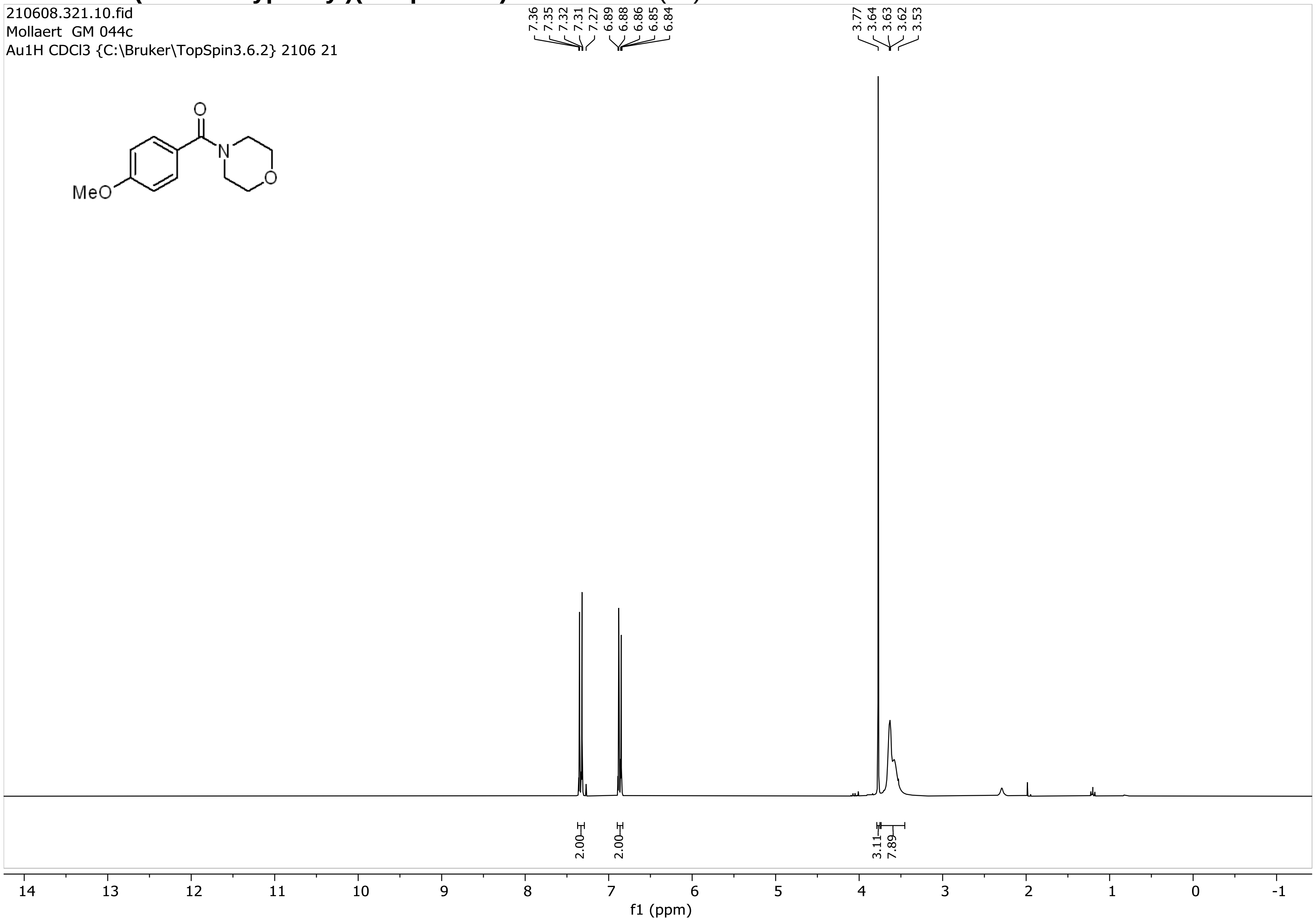

^{13}C NMR of (4-Methoxyphenyl)(morpholino)methanone (7c)

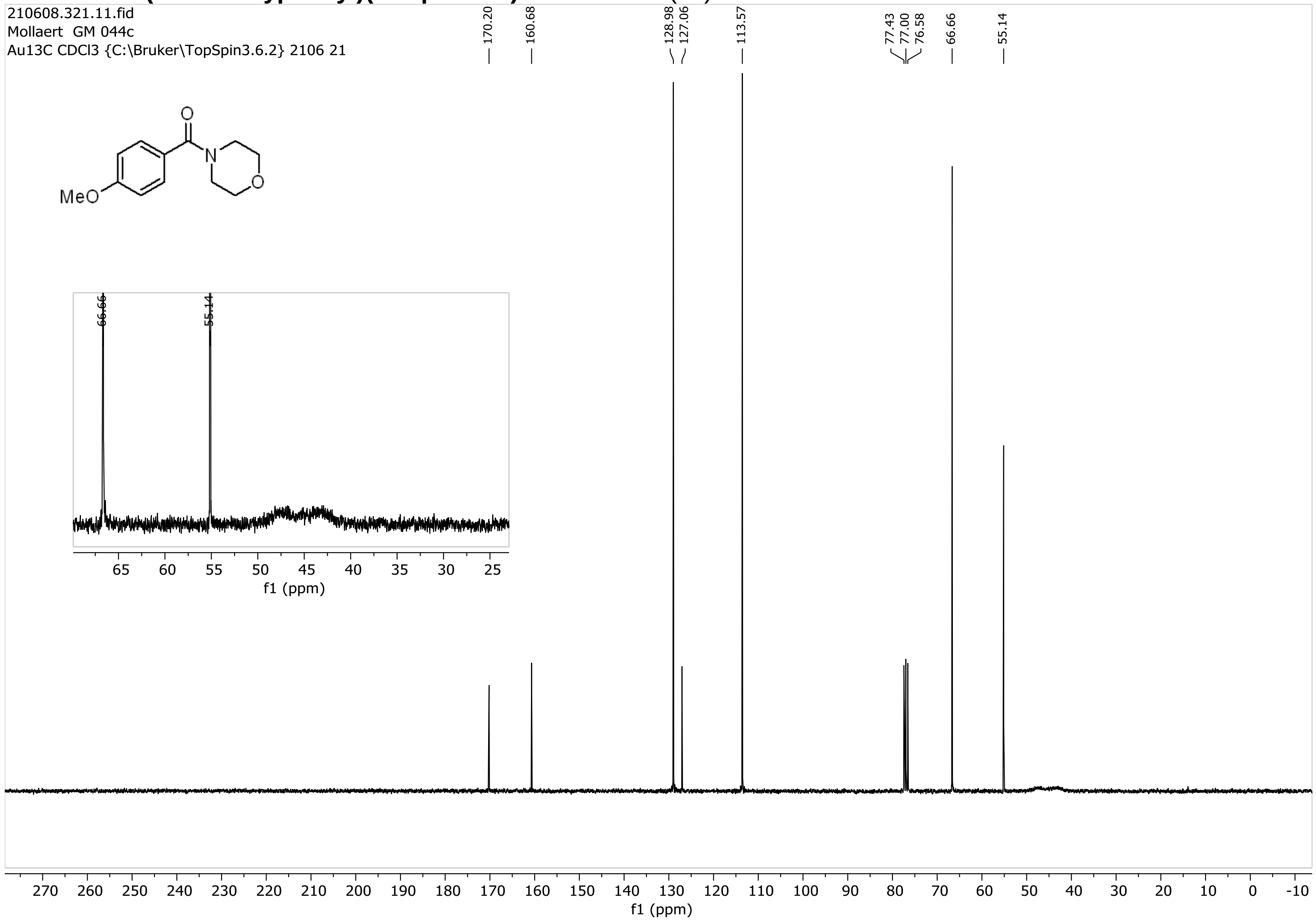

^{1}H NMR of 4-Methoxy-*N*,*N*-dipropylbenzamide (7d)

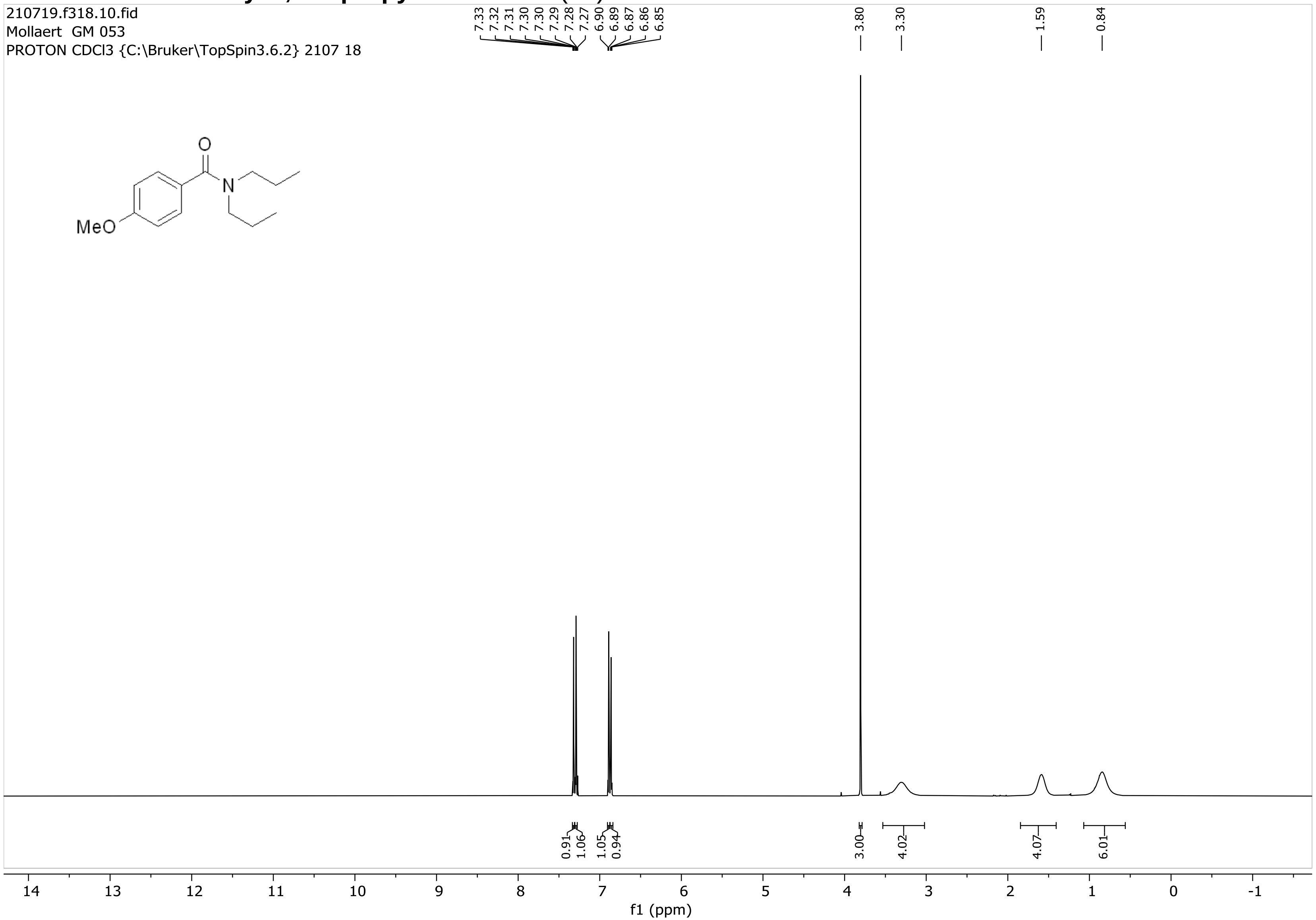

^{13}C NMR of 4-Methoxy-*N*,*N*-dipropylbenzamide (7d)

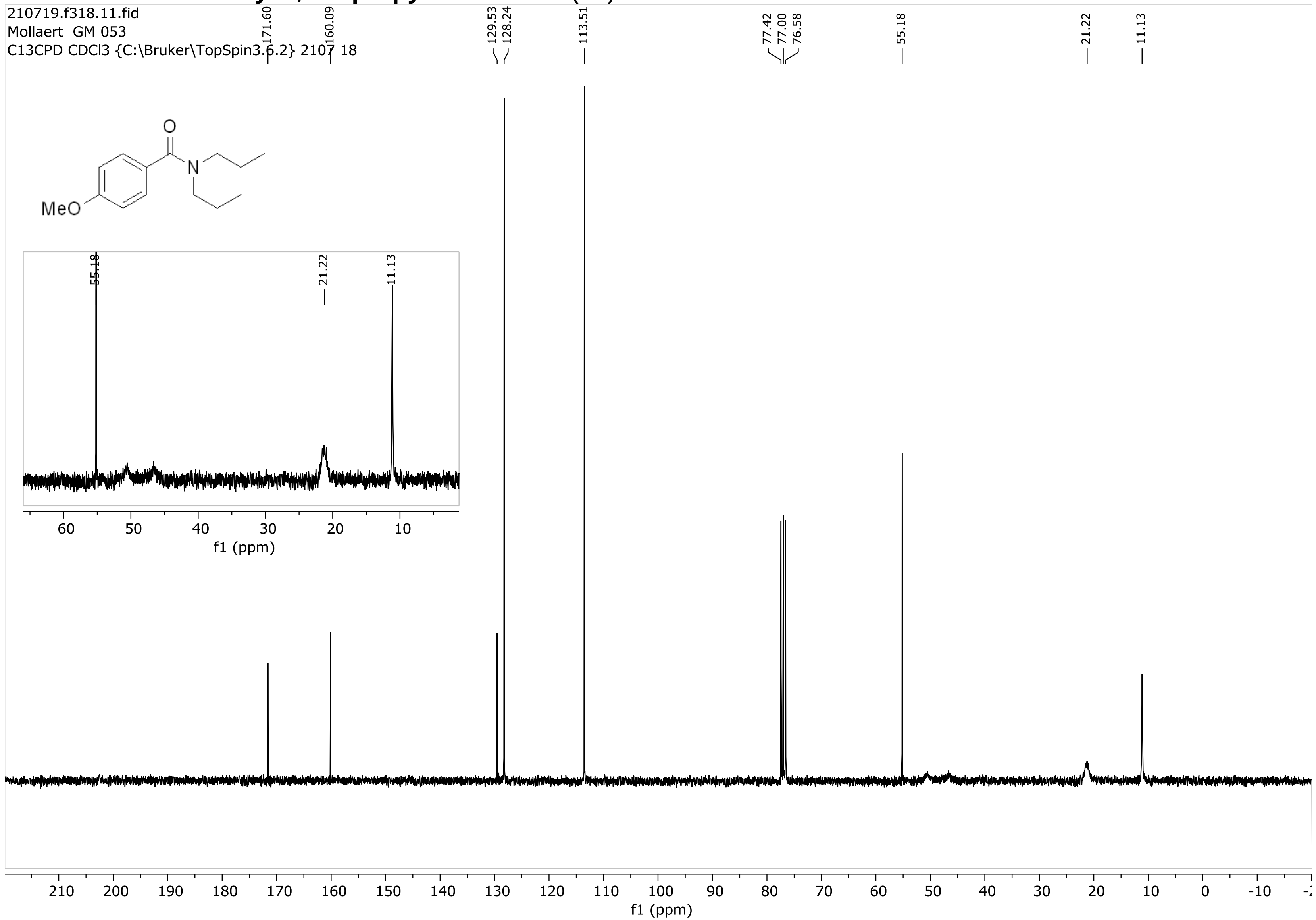

^{1}H NMR of *N*-Benzyl-4-methoxybenzamide (7e)

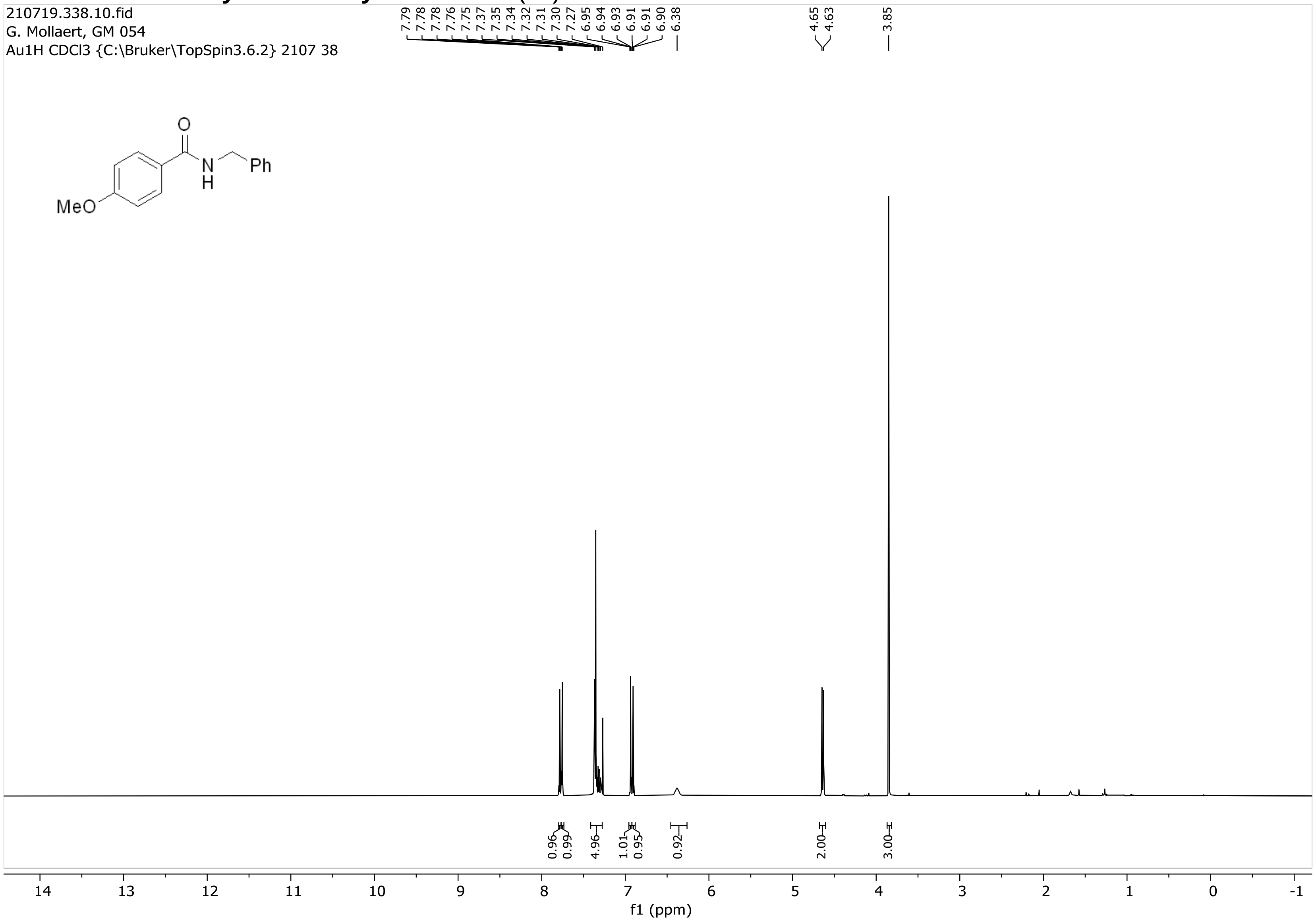

^{13}C NMR of *N*-Benzyl-4-methoxybenzamide (7e)

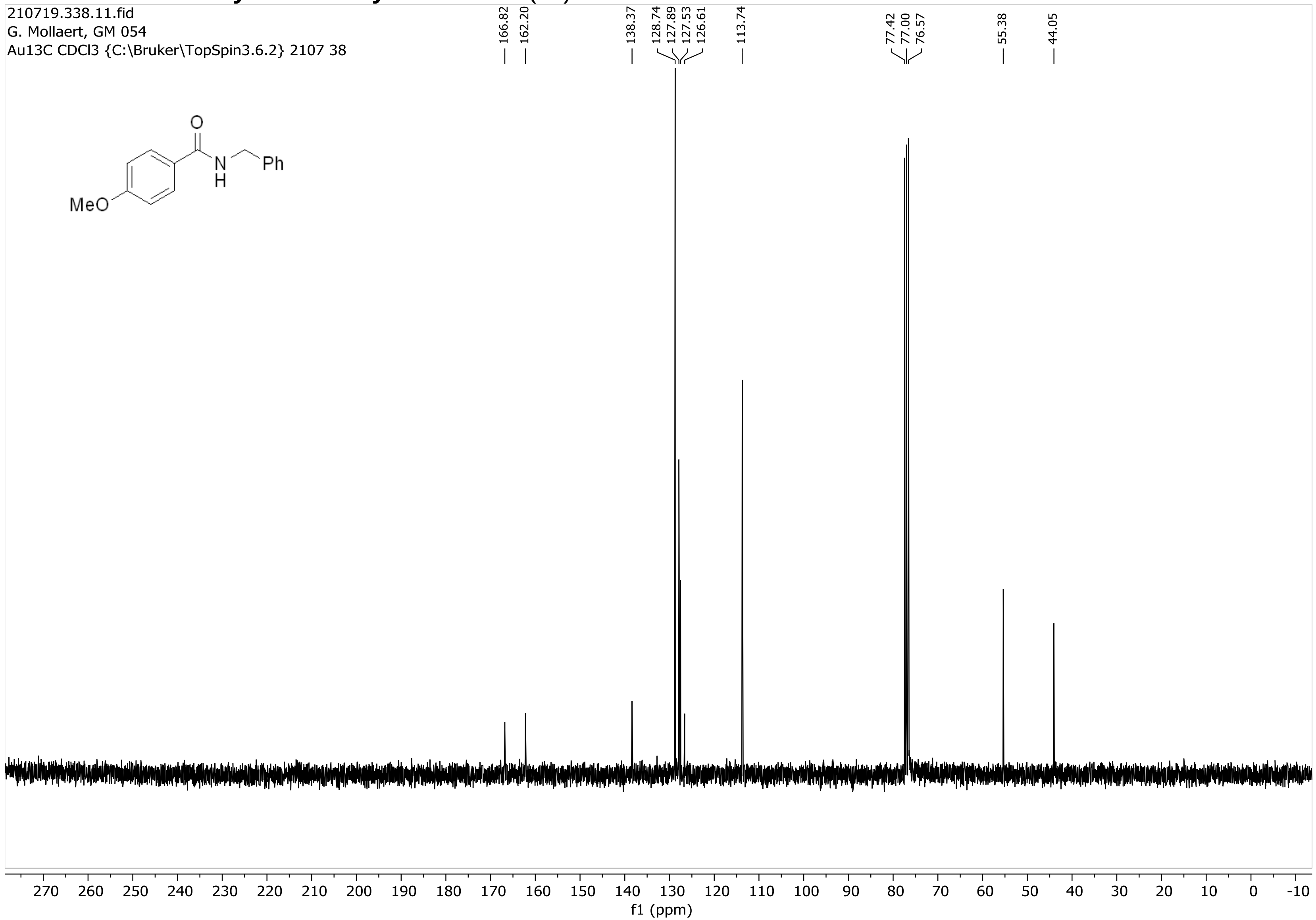

Supplementary References

1. Perkas, N., Gunawan, P., Amirian, G., Wang, Z., Zhong, Z. & Gedanken, A. The sonochemical approach improves the CuO–ZnO/TiO_2 catalyst for WGS reaction. *Phys. Chem. Chem. Phys.* **16**, 7521–7530 (2014).
2. Li, C., Wei, W., Fang, S., Wang, H., Zhang, Y., Gui, Y., & Chen, R. A novel CuO-nanotube/SnO_2 composite as the anode material for lithium ion batteries. *J. Power Sources* **195**, 2939–2944 (2010).
3. Eom, K., Kim, S., Lee, D. & Seo, H. Physicochemical interface effect in Cu_2O–ZnO heterojunction on photocurrent spectrum. *RSC Adv.* **5**, 103803–103810 (2015).
4. Staniuk, M., Zindel, D., van Beek, W., Hirsch, O., Kränzlin, N., Niederberger, M. & Koziej, D. Matching the organic and inorganic counterparts during nucleation and growth of copper-based nanoparticles–in situ spectroscopic studies. *Cryst. Eng. Comm*. **17**, 6962–6971 (2015).
5. Huang, Z., Cui, F., Kang, H., Chen, J., Zhang, X. & Xia, C. Highly dispersed silica-supported copper nanoparticles prepared by precipitation− gel method: a simple but efficient and stable catalyst for glycerol hydrogenolysis. *Chem. Mater.* **20**, 5090–5099 (2008).
6. Bond, G. C., Namijo, S. N. & Wakeman, J. S. Thermal analysis of catalyst precursors: Part 2. Influence of support and metal precursor on the reducibility of copper catalysts. *J. Mol. Catal.* **64**, 305–319 (1991).
7. Chang, F. W., Kuo, W. Y. & Lee, K. C. Dehydrogenation of ethanol over copper catalysts on rice husk ash prepared by incipient wetness impregnation. *Appl. Catal. A Gen.* **246**, 253-264 (2003).
8. Wang, Z., Liu, Q., Yu, J., Wu, T. & Wang, G. Surface structure and catalytic behavior of silica-supported copper catalysts prepared by impregnation and sol–gel methods. *Appl. Catal. A Gen.* **239**, 87–94 (2003).
